# ESRP1 exerts anti-tumor role by promoting ferroptosis in diffuse-type gastric cancer

**DOI:** 10.1186/s13046-025-03435-2

**Published:** 2025-06-18

**Authors:** Kang Xu, Hao Chen, Xingyu Zhu, Han Li, Xinyu Liu, Yaodong Sang, Xiaohan Wang, Xiaoling Cui, Baoshan Cai, Liang Shang, Wei Chong, Leping Li

**Affiliations:** 1 Department of Gastrointestinal Surgery, Shandong Provincial Hospital, Shandong First Medical University, Jinan, 250021 China; 2 Shandong Provincial Laboratory of Translational Medicine Engineering for Digestive Tumors, Shandong Provincial Hospital, Jinan, 250021 China; 3 Medical Science and Technology Innovation Center, Shandong First Medical University & Shandong Academy of Medical Sciences, Jinan, 250021 China; 4 Clinical Research Center of Shandong University, Clinical Epidemiology Unit, Qilu Hospital of Shandong University, Jinan, 250000 China; 5 Department of Epidemiology and Health Statistics, School of Public Health, Cheeloo College of Medicine, Shandong University, Jinan, 250000 China; 6 Department of General Surgery, The First Affiliated Hospital of Shandong First Medical University, Jinan, 250000 China

**Keywords:** Epithelial splicing regulatory protein 1, Ferroptosis, Diffuse-type gastric cancer, Tumor mutational burden, Metabolism, Therapeutic target

## Abstract

Diffuse-type gastric cancer (DGC) accounts for approximately one-third of gastric cancer cases, but it is clinically more aggressive and associated with poorer survival prognosis compared to intestinal-type gastric cancer (IGC). Our study revealed significantly reduced ESRP1 expression in DGC patients relative to IGC counterparts, with its high expression positively correlating with DGC patient prognosis. Both in vitro and in vivo experimental models demonstrated that ESRP1 has the ability to hinder the malignant progression of DGC. Mechanistically, ESRP1 interacts with DHCR7, a ferroptosis-promoting regulator, thereby upregulating DHCR7 expression and enhancing ferroptosis susceptibility in DGC cells. In DGC patients with high ESRP1 expression, ferroptosis-inducing therapy emerges as a promising treatment alternative. Moreover, for ESRP1-low DGC cases, we identified several potent small-molecule drugs. These findings collectively position ESRP1 as a potential therapeutic target for DGC intervention.

**Supplementary Information:**

The online version contains supplementary material available at 10.1186/s13046-025-03435-2.

## Introduction

Gastric cancer (GC) is the fifth most commonly diagnosed malignancy and the fifth leading cause of cancer-related mortality worldwide [1]. According to the Lauren classification, gastric adenocarcinoma is histologically stratified into two primary subtypes: intestinal-type and diffuse-type [2]. Clinically, the incidence of intestinal-type gastric cancer (IGC), the most common Lauren subtype, has been steadily declining, while the frequency of diffuse-type gastric cancer (DGC) is increasing and comprises approximately 30% of all GC cases [3–5]. Accumulating evidence indicates that DGC displays more aggressive biological behavior and is associated with poorer clinical outcomes compared to IGC [6–8]. Histopathologically, DGC is characterized by weak cell-cell cohesion, resulting in diffuse tumors along the gastric lining. Consequently, detecting DGC is difficult, and many patients are found to have advanced stages of the disease, which contributes to the poor prognosis and therapeutic failure of DGC [9]. Notably, molecular targeted therapy for specific patient populations has shown significant improvement in curative effects and prognosis [10–12]. Despite the fact that alterations in the molecular state of genes associated with gastric cancer are an important factor in treatment selection, few effective targets have been discovered thus far for DGC patients. Therefore, it is imperative to explore new molecular targets for the early diagnosis and treatment of patients with DGC.

Epithelial splicing regulatory protein 1 (ESRP1), an RNA-binding protein, serves as an epithelial cell-specific splicing factor that regulates alternative splicing of multiple genes, including *FGFR2*, *CD44*, *CTNND1*, and *ENAH*, which play critical roles in epithelial- mesenchymal transition (EMT) [13–15]. Increasing evidence has demonstrated that ESRP1 dysregulation is closely correlated with the tumorigenesis and progression of various types of cancers, including ovarian cancer [16], breast cancer [17], prostate cancer [18], colorectal cancer [19], lung cancer [20] and so forth. In gastric cancer, previous study has shown that 85% of patients presenting low ESRP1 expression were of diffuse type [21]. Another study revealed an inverse correlation between ESRP1 levels and metastatic potential, with significantly reduced ESRP1 expression observed in gastric cancer patients with lymph node or distant metastases compared to non-metastatic cases [22]. Additionally, ESRP1 plays a crucial role in the chemoresistance of multiple cancers and functions as a novel drug resistance marker molecule and a potential therapeutic target [17, 23, 24].These findings may highlight the clinical significance of ESRP1 in tumor progression and metastasis and offer a potential predictive system for evaluating outcomes in patients with gastric cancer. However, the role and specific regulatory mechanisms of ESRP1 in gastric cancer, particularly in DGC, remain uncertain.

Ferroptosis is a form of non-apoptotic cell death that depends on the accumulation of iron in cells and induced by toxic lipid peroxidation [25, 26]. Accumulating evidence suggests that ferroptosis is a critical mechanism for tumour suppression, exerting its antitumor effects through interactions with various tumor suppressor genes [27–29]. TP53, the most commonly mutated tumor suppressor gene, stands out as a prominent example linking ferroptosis to tumor suppression [29, 30]. P53 promotes ferroptosis by reducing the expression of SLC7A11 [31]. Similarly, the tumor suppressor AMER1 promotes ferroptosis susceptibility in colorectal cancer by facilitating ubiquitination-mediated degradation of SLC7A11 and ferritin light chain [32]. These mechanistic insights highlight ferroptosis induction as a promising therapeutic strategy.

In this study, we demonstrated significantly reduced ESRP1 expression in DGC compared to IGC. Functionally, ESRP1 suppressed DGC proliferation and metastatic dissemination, with its expression levels positively correlating with patient survival, consistent with a tumor-suppressive role. Importantly, we uncovered a previously unrecognized link between ESRP1 and ferroptosis susceptibility in DGC. Mechanistic investigations identified DHCR7 (a pro-ferroptotic regulator) as an ESRP1-interacting protein. ESRP1-mediated upregulation of DHCR7 enhanced ferroptosis sensitivity in DGC cells. These findings suggest that ESRP1 may represent an important therapeutic target for DGC.

## Methods and materials

### Data resources

Multi-omics data and clinical metadata of samples were retrospectively collected from publicly available datasets of The Cancer Genome Atlas (TCGA-STAD), Asian Cancer Research Group (ACRG) cohort (GSE62254), Singapore cohort (GSE15459), Korea University Guro Hospital (KUGH) cohort (GSE26899), Kosin University College of Medicine (KUCM) cohort (GSE26901), Beijing Cancer Hospital (BJCH) cohort (GSE57303), PKU cohort (PXD008840, ProteomeCentral Datasets), and Clinical Proteomic Tumor Analysis Consortium (CPTAC-STAD). The clinical and survival information of the included datasets was summarized in Supplementary Table S1.

### Identification of differentially expressed genes between DGC and IGC

The differentially expressed genes (DEGs) between DGC and IGC were analyzed across six public datasets, including TCGA, ACRG, Singapore, KUGH, KUCM, and BJCH cohort. We specifically selected the diffuse or intestinal cases based on clinical pathological annotations from these studies. Two-tailed Student’s t-test was employed to determine DEGs between DGC and IGC in each dataset, using log-normalized expression data. *p*-values were adjusted using Benjamini-Hochberg (BH) correction, and adjusted *p*-values < 0.05 were considered as significantly different.

### Comparison of genomic alterations

The somatic mutation and copy number variation (CNV) data were downloaded from the TCGA database (https://portal.gdc.cancer.gov/). Nonsynonymous mutations (including frameshift mutation, inflame mutation, missense mutation, nonsense mutation and splice site mutation) counts were recognized as tumor mutation burden (TMB). The significantly mutated genes (SMGs) were visualized using the waterfall function within the “Maftools” R package according to previous studies [33, 34].

### Deciphering mutational signature operative in the genome

The R package “Maftools” was used to extract mutational signatures from the TCGA genomic data. The Extract Signatures function based on Bayesian variant non-negative matrix factorization, factorized the mutation portrait matrix into two non-negative matrices “signatures” and “contributions,” where “signatures” represented mutational processes and “contributions” represented the corresponding mutational activities [35]. Specifically, the number of columns of matrix “signatures” indicated the number of extracted signatures and the rows indicated the 96 mutational contexts (i.e., C > G, C > A, C > T, T > C, T > A, T > G and combined their 50 and 30 adjacent bases). The Signature Enrichment function can automatically determine the optimal number of extracted mutational signatures and assign them to each sample based on the mutational activities. The extracted mutational portrait of CC was compared and annotated by cosine similarity analysis against the Catalogue of Somatic Mutations in Cancer (COSMIC) [36–38].

### Analysis of metabolic signature differences

We utilized the R package “IOBR” to explore differences in biological processes between the ESRP1-high and ESRP1-low subgroups. The ssGSEA algorithm was employed to calculate pathway activity scores for each sample. For each pathway, the Wilcoxon rank-sum test was applied to compare activity differences between the ESRP1-high and ESRP1-low groups, calculating significance *p*-values. Multiple hypothesis testing was adjusted using the Benjamini-Hochberg method, with significant differential metabolic pathways screened at a threshold of FDR < 0.05.

### ScRNA‑seq analysis and compare the enrichment score of the cell subpopulations

The processed GC single cell RNA sequencing (scRNA-seq) data, cell annotations, and matched bulk-RNA expression profile were obtained from Sun et al. study [39] (OMIX database, accession ID: OMIX001073). To ensure data homogeneity, we excluded samples derived from blood or Lauren intestinal subtypes, as well as those lacking paired bulk RNA-seq data. After filtration, 104,028 cells from 7 gastric cancer (GC) patients were retained for downstream analysis. Immune cell subpopulations were collected from Zeng et al. [40]. and their enrichment score were calculated using the AddModuleScore function in Seurat (v5.0). Based on ESRP1 median expression levels in bulk RNA-seq data, the 7 GC samples were stratified into high- and low- expression subgroups. Differential enrichment scores between these subgroups were assessed using the Wilcoxon rank-sum test.

### Cell culture

Human DGC cell lines MKN45 were kindly obtained from Key Laboratory for Experimental Teratology of the Ministry of Education, Department of Pathology, School of Basic Medical Sciences, Shandong University. The human DGC cell lines SUN484 was purchased from Meisen Chinese Tissue Culture Collections.MKN45 and SUN484 cells were cultured in RPMI-1640 medium (Gibco, USA) supplemented with 10% fetal bovine serum (FBS), and 1% penicillin/streptomycin (100U/mL, Solarbio, China) in a humidified atmosphere containing 5% CO_2_ at 37 °C.

### Plasmids, SiRNA and cell transfection

ESRP1 shRNA lentivirus, ESRP1 overexpressing lentivirus, and plasmids were purchased from GeneChem (Shanghai, China). Small interfering RNAs (siRNA) and negative control (NC) siRNAs were designed and chemically synthesized by Keyybio (Shandong, China). The sequences for siDHCR7: 5’-GGCCAAGACUCCACCUAUA-3’. Stable clones were maintained using puromycin. Transfections of siRNA were performed using Lipofectamine2000 Reagent.

### Quantitative real-time PCR

Total RNA was extracted by Trizol reagent (Vazyme, China). Then, reverse transcription was performed using a HiScript III RT SuperMix according to the manufacturer’s protocol (Vazyme, China). The ChamQ Universal SYBR qPCR Master Mix (Vazyme, China) was used in qPCR. All primers used in this study were listed below: GAPDH-F: GAGAAGTATGACAACAGCCTCAA, GAPDH-R: GCCATCACGCCACAGTTT, ESRP1-F: ATCTTAGCGGTGTCCCTCCA, ESRP1-R: TCCTGGCCTGGTCATTTGTG, DHCR7-F: GGGCTACTACATCTTCCGGG, DHCR7-R: TGTAGGAGCACTCGATGACC, GPX4-F: GAGGCAAGACCGAAGTAAACTAC, GPX4-R: CCGAACTGGTTACACGGGAA, MUC1-F: TGCCGCCGAAAGAACTACG, MUC1-R: TGGGGTATCGCTCATAGGAT, AKR1C3-F: GAGACAAACGATGGGTGGACC, AKR1C3-R: TGGAACTCAAAACCTGCACG, SLC7A11-F: TCTCCAAAGGAGGTTACCTGC, SLC7A11-R: AGACTCCCCTCAGTAAAGTGAC.

### Western blotting and antibodies

Total proteins of cells were extracted using RIPA lysate (Solarbio, Beijing, China). The BCA kit (Solarbio, Beijing, China) was used for protein concentration detection. Briefly, 10 µg loading protein was separated with 10% SDS-PAGE, and then transferred onto PVDF membranes. The membranes were blocked with 5% skim milk for 1 h and incubated at 4 °C overnight with primary antibody. Then the membranes were incubated with the corresponding species secondary antibody (Proteintech) for 1 h. Finally, ECL Kit (Solarbio, Beijing, China) was used for detection, and relative quantitative values of the bands were measured by ImageJ software (v.1.4.3.67). All antibodies used in this study were listed below: anti-ESRP1 (ab107278, Abcam, rabbit, 1:1000), anti-ESRP1(21045-1-AP, Proteintech, rabbit, 1:1000), anti-β-actin (20536-1-AP, Proteintech, rabbit, 1:2000), anti-GAPDH (60004-1-Ig, Proteintech, mouse, 1:5000), anti-Flag (66008-1-Ig, Proteintech, mouse, 1:5000), anti-DHCR7 (PA5-48204, Invitrogen, rabbit, 1:2000), anti-GPX4 (ab125066, Abcam, rabbit, 1:5000), anti-SLC7A11 (ab175186, Abcam, rabbit, 1:3000), anti-SLC3A2 (15193-1-AP, Proteintech, rabbit, 1:5000), anti-TFRC (10084-2-AP, Proteintech, rabbit, 1:1500), anti-PTGS2 (12282T, CST, rabbit, 1:1000), anti-VDAC2 (11663-1-AP, Proteintech, rabbit, 1:1500), anti-MUC1(CY5709, Abways, rabbit, 1:1000), anti-AKR1C3(CY8300, Abways, rabbit, 1:1000).

### Cell proliferation assays

Cell proliferation assays were performed with Cell Counting Kit-8 (DojinDo, Japan). Cells were seeded at a density of 3 × 10^3^ cells per well in the 96-well plate. CCK-8 solution was added and the absorbance value was measured at 450 nm after 2 h.

### Colony formation assay

Cells (1 × 10^3^cells/well) were seeded into 6-well plates and cultured for 10–14 days at 37 °C, and the culture medium was replaced with fresh medium every 3–4 days. Then, cells were washed using phosphate- buffered saline (PBS), fixed with 4% paraformaldehyde for 30 min at room temperature, and stained with 0.5% crystal violet for 30 min at room temperature. The number of colonies (containing > 50 cells) was observed and counted.

### Wound healing assay

For wound healing assay, cells were seeded into 6-well plate and ensured 100% cell fusion overnight. After the cells reached the fusion density, the 200 µL tip was used to make a vertical line scratch, and after the scratch, the cells were washed away with PBS, and the serum-free medium was replaced to continue the culture, and the cells were photographed through a microscope at 0 h, 24 h, and 48 h, respectively, and the mobility was measured and calculated by using ImageJ software (v.1.4.3.67).

### Cell migration and invasion assay

For migration and invasion analysis, cells (200 µL of serum-free medium) were seeded onto 8-mm Pore Transwell Inserts (Corning) coated with Matrigel for invasion assay, or without Matrigel for migration assay. Lower chambers were filled with 10% FBS medium (600 µL). Following a 24 h incubation at 37℃, cells on the Transwell Inserts were then fixed with 4% paraformaldehyde for 30 min. Next, fixed cells were stained with hematoxylin solution for 30 min. Then microphotograms of the cells migrated onto the lower side of the filter were imaged using a microscope.

### Subcutaneous xenograft model

A total of 5 × 10^6^ MKN45-vector/MKN45-ESRP1 cells in 100 µL PBS were injected subcutaneously into the right axilla of nude mice. After tumor formation, tumor size was measured every 2–3 days along both vertical axes using vernier calipers. After 3 weeks, mouse live imaging was performed. Three weeks after inoculation, mice were euthanized, tumors were isolated, weighed, and photographed. The tumor volume was calculated using the formula (volume = length × width^2^ × 0.5).

### Mouse liver metastasis model

To establish liver metastasis models, 2 × 10^6^ MKN45-vector or MKN45-ESRP1 cells were suspended in 100 µL of sterile PBS and injected into the spleen of nude mice. At 6 weeks post-injection, mice were humanely euthanized. Livers were harvested, fixed in 4% paraformaldehyde for 24 h, and paraffin-embedded. Tissue sections were prepared for H&E staining to evaluate metastatic tumor burden through histopathological analysis.

### Mouse lung metastasis model

To evaluate the lung metastatic potential of GC cells in vivo, 1 × 10^6^ cells MKN45-vector/MKN45-ESRP1 cells were resuspended in 100 µL of sterile PBS and injected into the tail vein of the nude mice. Six weeks post-cell injection, the nude mice were euthanized, and their lung tissues were harvested for histological H&E analysis to quantify the number of metastatic tumor nodules.

### Lymph node metastasis model

For establishment of lymph node metastasis model, BALB/c-nude mice (4–6 weeks old, 18–20 g body weight) were selected for the study. The left-side foot pad of each mouse was disinfected with 75% ethanol. ESRP1-overexpressing MKN45 gastric cancer cells and their control counterparts were suspended at a density of 2.5 × 10⁷ cells/mL in sterile PBS. A total of 5 × 10⁵ cells (in 20 µL volume) were subcutaneously injected into the disinfected left footpad using an insulin syringe. Post-injection, mice were monitored for tumor growth and lymph node metastasis progression. Four weeks post-cell injection, nude mice were euthanized, and both the footpad xenografted tumors and popliteal lymph node were resected, sectioned and histologically examined by H&E staining.

### Cell viability and cell death assay

Cell viability was performed with Cell Counting Kit-8. Cell death was measured by propidium iodide (PI) staining using a fluorescence microscope. Cells were seeded at a density of 30–40% per well into 12-well plates. Overnight, cells were treated with RSL3(10 µM) or RSL3(10 µM) and Fer-1(5 µM) at indicated concentrations for 24 h. To identify dead cells, PI (Final concentration: 5 µg/mL) was added to the medium for an additional 30 min. Subsequently, PI-positive dead cells were analyzed using a fluorescence microscope through TRITC fluorescent channel.

### Reactive oxygen species (ROS) measurement

ROS detection Kit (Solarbio, China) were used to analyze the cellular ROS level according to the manufacturer’s protocol. Briefly, cells were seeded into a 96-well plate at a density of 1 × 10^5^ cells per well and incubated overnight. Subsequently, 200 µL of staining solution with a concentration of 10 µM was added to each well, followed by incubation at 37 °C for 30 min. After removing the staining solution, the wells were washed twice with PBS, and then the cells were observed under a fluorescence microscope.

### Measurements of malondialdehyde (MDA)

Lipid peroxidation MDA assay kit (Beyotime, China) was used to assess the malondialdehyde (MDA) level according to the manufacturer’s protocol. Briefly, cells that received various treatments were lysed to prepare supernatants. After that, the supernatants were reacted with 200 µL of MDA reaction solution at 100 °C for 15 min. Then, MDA levels were measured at 532 nm using a microplate reader.

### Glutathione (GSH) assay

For GSH analysis, cell counting was performed after digesting the cells using trypsin, and an equal number of cells were collected. GSH detection Kit (Solarbio, China) were used to analyze the level of GSH in GC cells according to the manufacturer’s protocol.

### Transmission Electron Microscope (TEM)

Cells were subjected to the indicated treatment and then underwent centrifugation at 1000 rpm for 5 min following trypsinization. Afterward, the cells were fixed with 2.5% glutaraldehyde at 4 °C for a duration of 2 h. Subsequent to fixation, the cells were postfixed with 1% osmium tetroxide under the same temperature conditions for 1 h. The cells were then dehydrated using a series of alcohol and acetone solutions of increasing concentration. Following dehydration, the cells were embedded. TEM images were captured using Electron Microscope.

### Immunofluorescence

A total of 1 × 10^5^ MKN45 cells were seeded in 24-well plates and allowed to grow overnight before treatment. The cells were fixed with 4% PFA for 30 min at room temperature and permeabilized on ice with 0.2% Triton X-100 in PBS buffer for 10 min. The cells were blocked in PBS containing 3% BSA for 1 h at room temperature and then incubated with the appropriate primary antibody (1:200 dilution) for 2 h at room temperature. Next, the cells were incubated with a FITC- or Texas Red conjugated secondary antibody at room temperature for 1 h. Nuclei were stained with DAPI for 10 min. Images were acquired using a fluorescence microscope.

### Co-immunoprecipitation (Co-IP)

Cells are lysed in IP buffer with the appropriate protease inhibitor. Cell lysates were incubated overnight in a 4 °C rotary incubator with antibodies and then co-incubated with protein A/G magnetic beads overnight in a 4 °C rotary incubator. The magnetic beads were washed with PBST and boiled in 1× SDS-loading buffer at 100 °C for 5 min, and then the binding proteins were analyzed by Western blotting.

### RNA sequencing (RNA-seq) analysis

The RNA of Vector/MKN45, ESRP1/MKN45 cells was extracted using an RNA extraction reagent kit (Vazyme, China). Three independent samples (biological replicates) were established for each group. The RNA sequencing library was prepared and sequenced on an Illumina Novaseq 6000 platform (Majorbio, China) by Illumina sequencing. Differential expression analysis was performed using the ‘DESeq2’ package.

### Drug sensitivity analyses

We utilized the drug sensitivity data of the Genomics of Drug Sensitivity in Cancer (GDSC) and Profiling Relative Inhibition Simultaneously in Mixtures (PRISM) to explore the potential therapeutic agents [41]. Correlation analysis was performed between ESRP1 expression and drug sensitivity in GC cell lines. Gene dependency screening system (CERES and RNAi) and drug sensitivity database (GDSC1 and PRISM) were accessed from the dependency map (DepMap) portal (https://depmap.org/portal/). CCK-8 and colony formation assays were performed for in vitro validation of candidate drugs. For CCK-8 assay, cells were seeded in 96- well plates (3000 cells/well) and treated with candidate drugs (0, 0.01, 0.1, 1, 10, 100 µM) for 24 h. Cell viability was quantified using the CCK-8 kit measuring absorbance at 450 nm. Half-maximal inhibitory concentration (IC50) values were calculated using GraphPad Prism (v.10.0.1). For colony formation assay, cells were seeded in 6-well plates (1000 cells/well) and exposed to IC50 doses of candidate drugs for 10–14 days. Colonies were fixed with 4% paraformaldehyde, stained with 0.5% crystal violet, and counted (> 50 cells/colony).

### Statistical analysis

Statistical analysis was performed using R software (v.4.4.2). For variables that follow a normal distribution, Student’s t-test was utilized for comparing two groups, while one-way ANOVA was employed for comparing more than two groups. In cases where the normal distribution assumption did not hold, the Wilcoxon ranksum test or Kruskal-Wallis test was used as an alternative nonparametric method. R survival package was used for survival analysis, and the survival rate of each group was tested by Log-rank test hypothesis. Cox proportional hazards model was used to analyze the association between the ESRP1 and patient prognosis. Fisher’s exact test was used to analyze the mutation frequency between ESRP1 subgroup and gene mutations, and Spearman analysis was used to calculate the correlation coefficient. The statistical significance was set to *p* < 0.05.

## Results

### ESRP1 expression is positively correlated with the prognostic of DGC patients

**Fig. 1:**
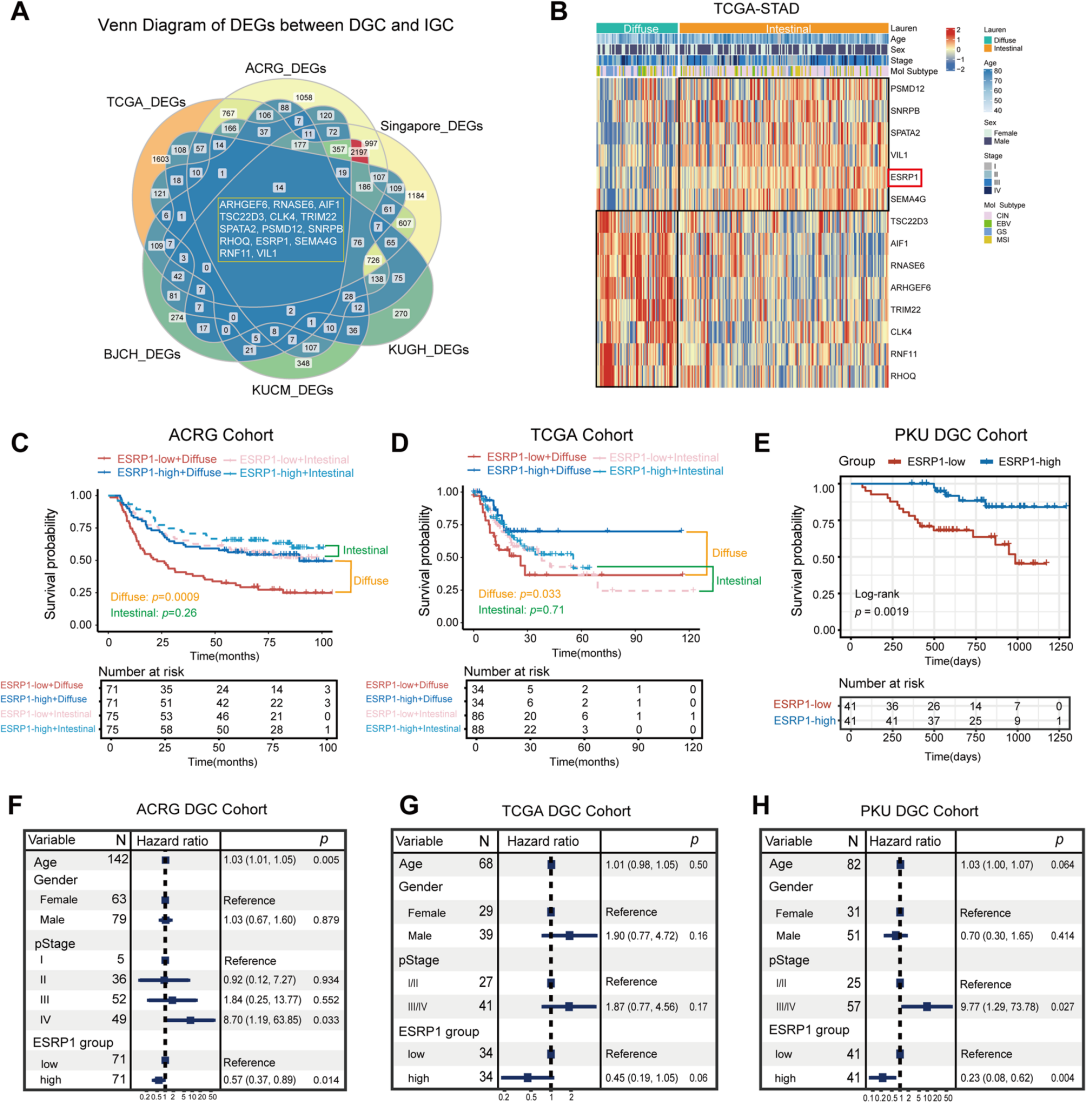
ESRP1 expression correlates with DGC prognosis. (**A**) Venn diagram of DEGs between DGC and IGC. (**B**) Heatmap of 14 overlapped genes between DGC and IGC in the TCGA cohort. (**C**) Kaplan-Meier curves of overall survival classified by ESRP1 median expression in the ACRG cohort, TCGA cohort (**D**), and PKU DGC cohort (**E**). (**F**) Forest plot representation of the multivariate Cox regression model delineates the association between ESRP1 and prognosis in the ACRG DGC cohort, TCGA DGC cohort (**G**), and PKU DGC cohort (**H**)

Previous studies have demonstrated that DGC patients show significantly worse clinical outcomes than those with IGC, with a higher propensity for recurrence and metastasis [6–8]. To identify potentially important genes related to the initiation and progression of DGC, we analyzed RNA sequencing data and microarray from public datasets to compare the DEGs between DGC and IGC. By intersecting the DEGs from six datasets (TCGA cohort, ACRG cohort, Singapore cohort, KUGH cohort, KUCM cohort, and BJCH cohort), we identified a total of 14 overlapped genes, namely *ARHGEF6*, *RNASE6*, *AIF1*, *TSC22D3*, *CLK4*, *TRIM22*, *SPATA2*, *PSMD12*, *SNRPB*, *RHOQ*, *ESRP1*, *SEMA4G*, *RNF11*, and *VIL1*(Fig. 1A, Table S2). Among these 14 genes, *TSC22D3*, *AIF1*, *RNASE6*, *ARHGEF6*, *TRIM22*, *CLK4*, *RNF11*, and *RHOQ* were significantly upregulated in DGC, while *PSMD12*, *SNRPB*, *SPATA2*, *VIL1*, *ESRP1*, and *SEMA4G* were significantly downregulated in DGC (Fig. 1B). We then performed a survival analysis on these 14 overlapping DEG to identify genes with significant prognostic implications using Kaplan-Meier Plotter, which revealed that only *RHOQ* (HR:1.51[1.07–2.12], *p* = 0.018) and *ESRP1* (HR:0.63[0.45–0.89], *p* = 0.0086) were significantly associated with DGC patient survival (Supplementary Fig. S1A-N). Additionally, through literature review, we have learned that ESRP1, as an epithelial-specific RNA-binding protein, can regulate tumor invasion and metastasis by influencing the EMT process. Consequently, we have selected ESRP1 as a candidate gene.

To further evaluate the relationship between ESRP1 expression levels and the prognosis of patients with DGC, we stratified gastric cancer patients from gastric cancer public datasets into ESRP1-high and ESRP1-low expression subgroups based on the median cut-off value of ESRP1 expression. Kaplan-Meier survival curves revealed that DGC patients with high ESRP1 expression exhibited better overall survival (OS) than those with low expression in the ACRG cohort (*p* = 0.0009, Fig. 1C) and the TCGA cohort (*p* = 0.033, Fig. 1D). However, there was no difference in prognosis between the ESRP1-high expression group and the ESRP1-low expression group in IGC patients from the ACRG cohort (*p* = 0.26, Fig. 1C), and the TCGA cohort (*p* = 0.71, Fig. 1D), suggesting that the relationship between ESRP1 expression levels and the prognosis of DGC may depend on the heterogeneity of histopathological types. Additionally, we obtained similar results in the PKU cohort, a proteomic dataset from 84 DGC patients with paired tumor and nearby tissue [6] (*p* = 0.0019, Fig. 1E). Multivariate analysis, which included age, gender, pathological stage (pStage), and ESRP1 expression, demonstrated that ESRP1 high expression was an independent prognostic factor for favorable OS in the ACRG diffuse-type cohort (HR: 0.57, 95% CI: 0.37–0.89, *p* = 0.014, Fig. 1F), the TCGA diffuse-type cohort (HR: 0.45, 95% CI: 0.19–1.05, *p* = 0.06, Fig. 1G), and the PKU cohort (HR: 0.23, 95% CI: 0.08–0.62, *p* = 0.004, Fig. 1H). Collectively, these findings indicate that ESRP1 plays a crucial role in DGC development and may serve as a potential prognostic biomarker for DGC.

### ESRP1 inhibits DGC cells proliferation, migration, and invasion in vitro and in vivo

**Fig. 2:**
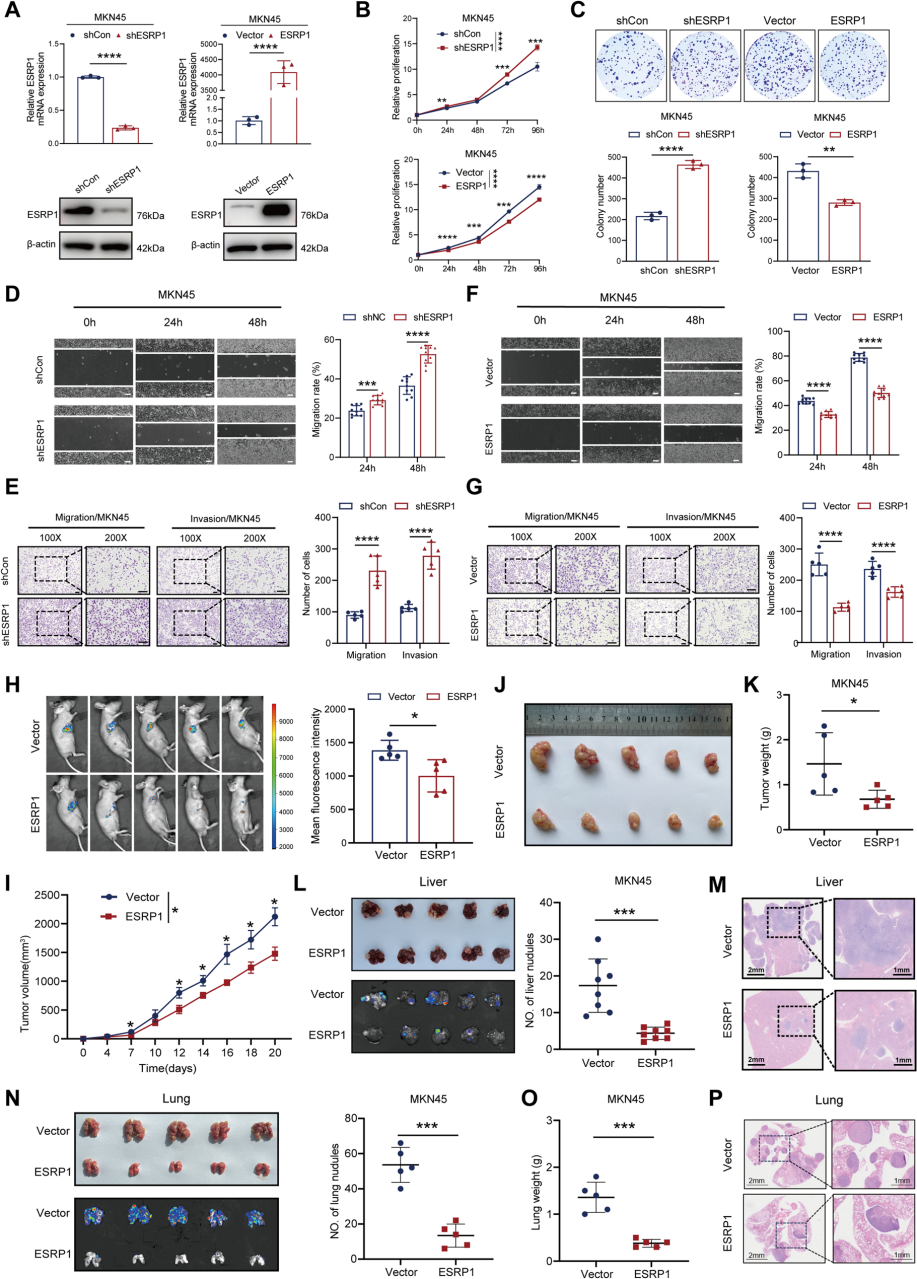
ESRP1 inhibits the malignant progression of DGC in vitro and in vivo. (**A**) The knockdown and overexpression efficiencies of ESRP1 in MKN45 cells were detected by qPCR combined with Western blotting. (**B**, **C**) Cell proliferation and colony formation ability of MKN45 cells with shCon (negative control), shESRP1 (ESRP1 knockdown), Vector (Vector control), ESRP1 (ESRP1 overexpression). (**D**, **E**) Wound healing and Transwell assays were performed to detect the migration and invasion of shCon and shESRP1 transfected MKN45 cells (scale bar: 100 μm). (**F**, **G**) Wound healing and Transwell assays were performed to detect the migration and invasion of overexpression-ESRP1 and vector-transfected MKN45 cells (scale bar: 100 μm). (**H**-**K**) Representative images of xenograft tumors in nude mice (*n* = 5 mice) and statistical analyses of tumor volumes and tumor weights in the different groups (*n* = 5 mice). (**L**) Representative images and quantification of metastatic liver nodules in the different groups (*n* = 8 mice). (**M**) Representative images of H&E staining of metastatic liver nodules in the ESRP1-overexpression and control group (left scale bar: 2 mm; right scale bar: 1 mm). (**N**) Representative images and quantification of metastatic lung nodules in the different groups (*n* = 5 mice). (**O**)Lung weight in the ESRP1-overexpression (*n* = 5 mice) and control group (*n* = 5 mice). (**P**) Representative images of H&E staining of metastatic lung nodules in the ESRP1-overexpression and control group (left scale bar: 2 mm; right scale bar: 1 mm). The error bars indicate the mean ± SD.*p < 0.05; **p < 0.01; ***p < 0.001, ****p < 0.0001

To explore the biological roles of ESRP1 in DGC, diffuse-type gastric cancer cell lines MKN45 and SNU484 were chosen for further functional experiments. We first constructed ESRP1-knockdown MKN45 cell lines, ESRP1-overexpresion MKN45 cell lines and ESRP1-overexpression SNU484 cell lines. The knockdown and overexpression efficiencies of ESRP1 were verified by both qPCR and western blotting (Fig. 2A and Fig. S2A, B). Cell Counting Kit-8 (CCK-8) assay and colony formation assays indicated that ESRP1 overexpression suppressed cell proliferation and colony formation (Fig. 2B, C and Fig. S2C, D), while ESRP1 knockdown exerted the opposite effect (Fig. 2B, C). Additionally, wound healing and Transwell assay indicated that silence of ESRP1 promoted the invasive and migrative ability in MKN45 cells (Fig. 2D, E), while overexpressing ESRP1 suppressed tumor invasion and migration in MKN45 cells and SUN484 cells (Fig. 2F, G and Fig. S2E, F). To further validate the role of ESRP1 in vivo, we established the subcutaneous xenograft tumor model and three metastasis models in nude mice. Consistent with the in vitro result, the tumor burden in ESRP1-overexpression group was reduced compared to the control group, suggesting that upregulated ESRP1 expression effectively inhabited tumor growth in nude mice (Fig. 2H-K). In addition, there were fewer liver metastasis nodules in ESRP1 overexpression group compared with the control group (Fig. 2L, M). Similarly, the ESRP1-overexpressing group exhibited a significantly lower number of metastatic tumor nodules in the lungs compared to the control group (Fig. 2N, P). Notably, lung weight was markedly reduced in ESRP1-overexpressing mice relative to their control counterparts (Fig. 2O). Additionally, we established a popliteal lymph node metastasis model via footpad injection in nude mice. The ESRP1-overexpressing group exhibited significantly smaller popliteal lymph nodes compared to controls (Fig. S2G-J), along with reduced volume and lighter weight of footpad primary tumors (Fig. S2K-N). To sum up, these results indicate that ESRP1 may play a tumor suppressive role in DGC progression.

### Comparison of genetic variations and mutation signatures of ESRP1 subgroups

**Fig. 3:**
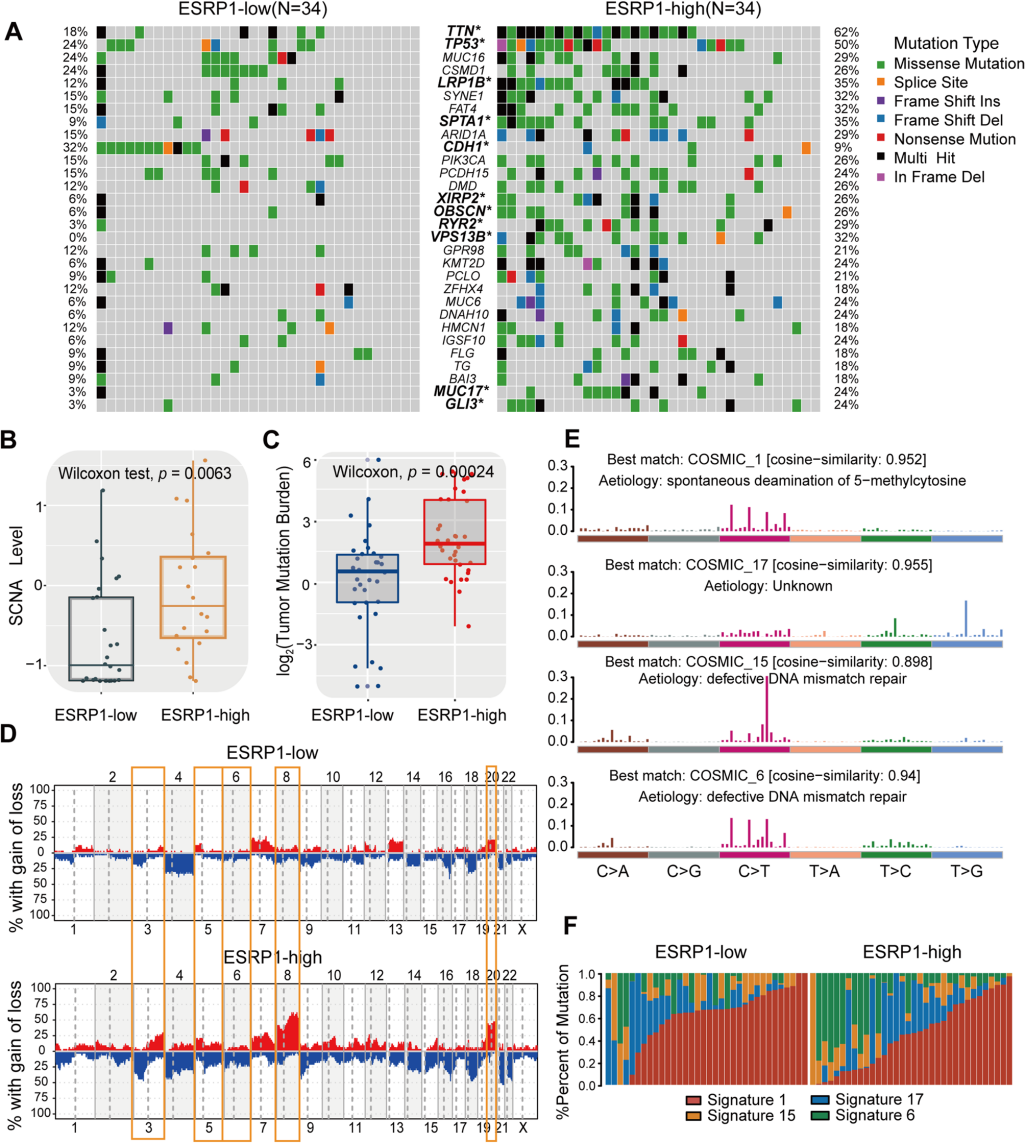
Comparison of genomic alterations between ESRP1-low expression group and -high expression group in the TCGA cohort. (**A**) Mutational landscape between ESRP1-low expression group and ESRP1-high expression group. (**B**) Comparison of SCNA between two groups. (**C**) Comparison of TMB between two groups. (**D**) A frequency of copy number gain and loss of chromosomes between ESRP1-low and -high expression group. (**E**)The mutational activities of corresponding extracted mutational signatures (signature1,17,15,6, named as COSMIC database). The trinucleotide base mutation types were on the x axes, whereas y axes showed the percentage of mutations in the signature attributed to each mutation type. (**F**) Distribution of mutational counts attributed to corresponding mutational signatures in different ESRP1 subgroups.

To investigate the differences of genetic mutations between ESRP1-low expression group and -high expression group, we analyzed somatic mutational profiles of 68 diffuse-type gastric cancer patients from TCGA. The mutational landscapes and the top 30 genes with the highest mutation frequencies in the two subgroups are presented in Fig. 3A. Among these, 11 genes exhibited significantly different mutation frequency between two subgroups, including *TTN*, *TP53*, *LRP1B*, *SPTA1*, *CDH1*, *XIRP2*, *OBSCN*, *RYR2*, *VPS13B*, *MUC17* and *GLI3* (Fig. 3A and Fig. S3A). Notably, only *CDH1* had higher mutation rates in the ESRP1-low expression subgroup compared to the ESRP1-high expression subgroup (Fig. 3A and Fig. S3A). In addition, patients with ESRP1-high expression exhibited higher SCNA and TMB compared with those in ESRP1-low expression group (Fig. 3B, C). At the chromosomal level, the copy number variation (both gain and loss) of the ESRP1-high group was relatively higher than the ESRP1-low group and the alterations were mainly concentrated on chr3, chr5, chr6, chr8 and chr20 (Fig. 3D). Of note, ESRP1-high group showed higher frequencies of CNV amplification on 8q24.21, 8q22.2, 8q21.11, 8p23.1; conversely, higher probabilities of CNV deletions on 3p14.2 (Fig. S3B). Previous studies had shown that the amplification of chromosome 8 is closely related to the occurrence and development of gastric cancer [42, 43]. For example, *CLDN23* gene is mapped to human chromosome 8p23.1 and played tumor suppressor effect in gastric cancer [43].

To gain further insights into the mutational process operative in patients with DGC, we delineated the mutation signatures from the mutational profile. The number of single-nucleotide variants (SNV) in the matrix of 96 possible mutations occurring in a trinucleotide context in each DGC sample were counted and found that the predominant mutations were characterized by the C > T transitions at Ap*C*pN trinucleotide sites (Fig. S3C). Subsequently, four mutation signatures were extracted from the genomic data of the TCGA cohort and annotated them according to the Catalog of Somatic Mutations in Cancer (COSMIC) signature nomenclature (Fig. 3E, F and Fig. S3D). We observed that the signature 1 consisted largely of C > T transition that was associated with spontaneous deamination of 5-methylcytosine. The signature 17 was associated with T > G for some unknown reasons. The signature 15 and signature 6 was associated with C > T transition due to defective DNA mismatch repair (Fig. 3E). Moreover, we found that mutational activities of signature 6 were significantly elevated in the ESRP1-high expression subgroup, while the signature 1 increased significantly in the ESRP1-low expression subgroup (Fig. 3F).

### Comparison of clinical characteristics and molecular processes of ESRP1 subgroups

**Fig. 4:**
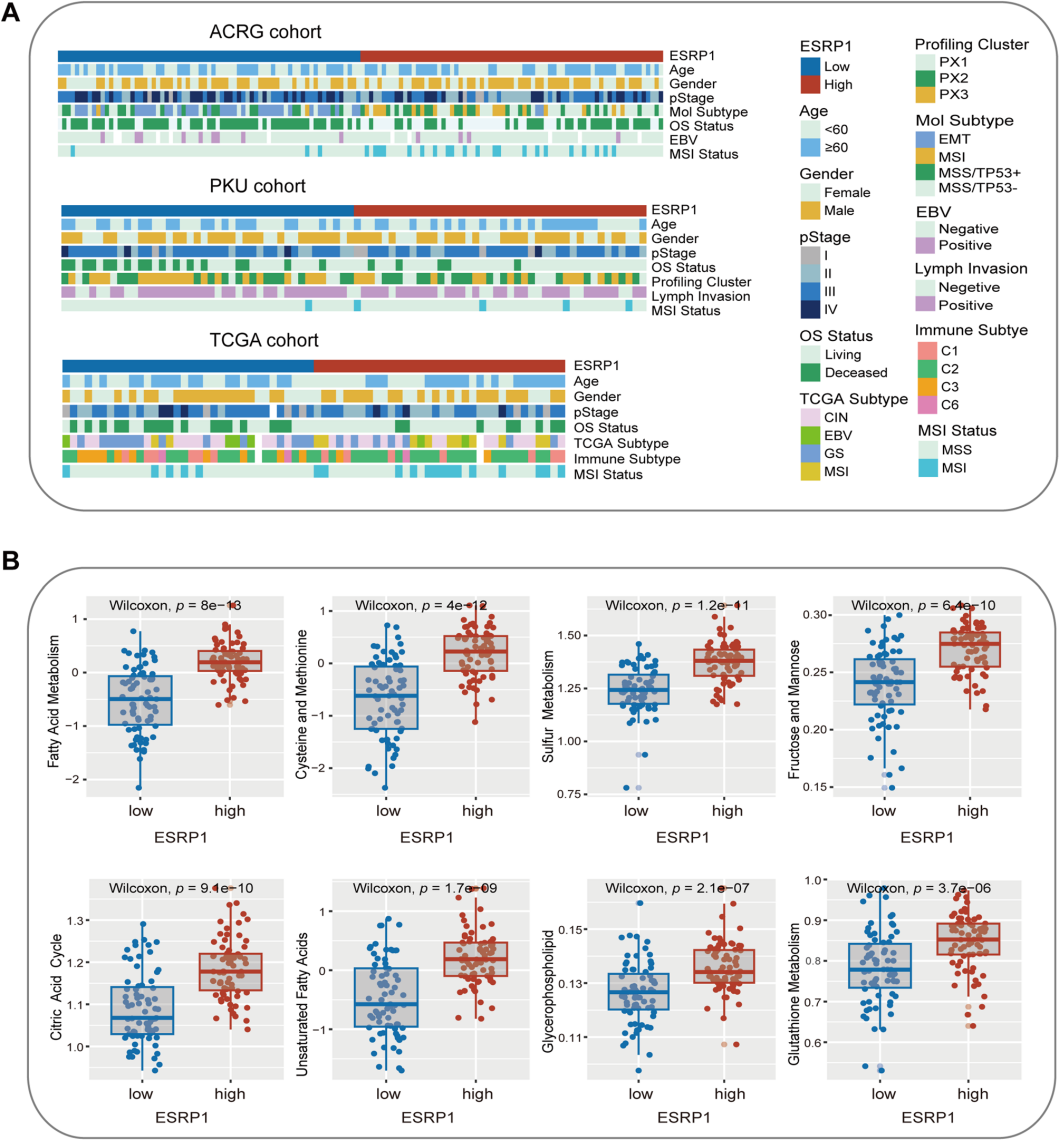
Comparison of clinical characteristics and molecular processes. (**A**) The relationship of clinical features and molecular subtypes among ESRP1-low and -high expression group in DGC from the ACRG cohort, PKU cohort, and TCGA cohort. (**B**) Comparison of metabolism-related differences between two subgroups in the ACRG cohort.

Furtherly, the relationships of ESRP1 expression level with clinical characteristics and molecular subtypes were explored in DGC patients. The EMT subtype (nomenclature by ACRG), genomically stable (GS) subtype (TCGA-defined), and invasion subtype (CPTAC-defined) were significantly enriched in ESRP1-low expression subgroup (Fig. 4A and Fig. S4A). These subtypes have a worse prognosis, which aligns with our findings. Interestingly, the MSI tumors in the ACRG cohort were markedly clustered into ESRP1-high expression subgroup (Fig. 4A). Similar results were ascertained in the PKU cohort and the TCGA cohort, which may be associated with a better survival outcome (Fig. 4A). In addition, the ESRP1-high subgroup was mainly characterized by metabolism-related integrated subtype and C2 immune subtype (Fig. S4A), then we further analyzed metabolic and immunological differences among the two subgroups. Fatty acid metabolism, glycerophospholipid metabolism, citric acid cycle, cysteine and methionine metabolism, sulfur metabolism, and glutathione metabolism were enriched in ESRP1-high expression subgroup (Fig. 4B). Single-cell technologies provide unprecedented mechanistic insights into tumor-stroma interactions and potential therapeutic vulnerabilities by enabling high-resolution dissection of cellular heterogeneity [33]. Therefore, we further utilized the scRNA-seq data for immune infiltration analysis among the ESRP1 subgroups. The results showed that 104,028 cells clustered into ten major cell types (Fig. S4B, C). Notably, the ESRP1 low subgroup exhibited substantial enrichment of immunosuppressive elements including cancer-associated fibroblasts (CAFs), macrophages, myeloid-derived suppressor cells (MDSCs), and regulatory T cells (Tregs). Conversely, ESRP1 high subgroup demonstrated preferential accumulation of tumor-reactive lymphocytes such as effector memory T cells (Tem), central memory T cells (Tcm), and natural killer (NK) cells. This immune landscape aligns with clinical outcomes, corroborating the adverse prognostic implications of ESRP1 deficiency (Fig. S4D).

### ESRP1 enhances sensitivity to ferroptosis of DGC cell

**Fig. 5:**
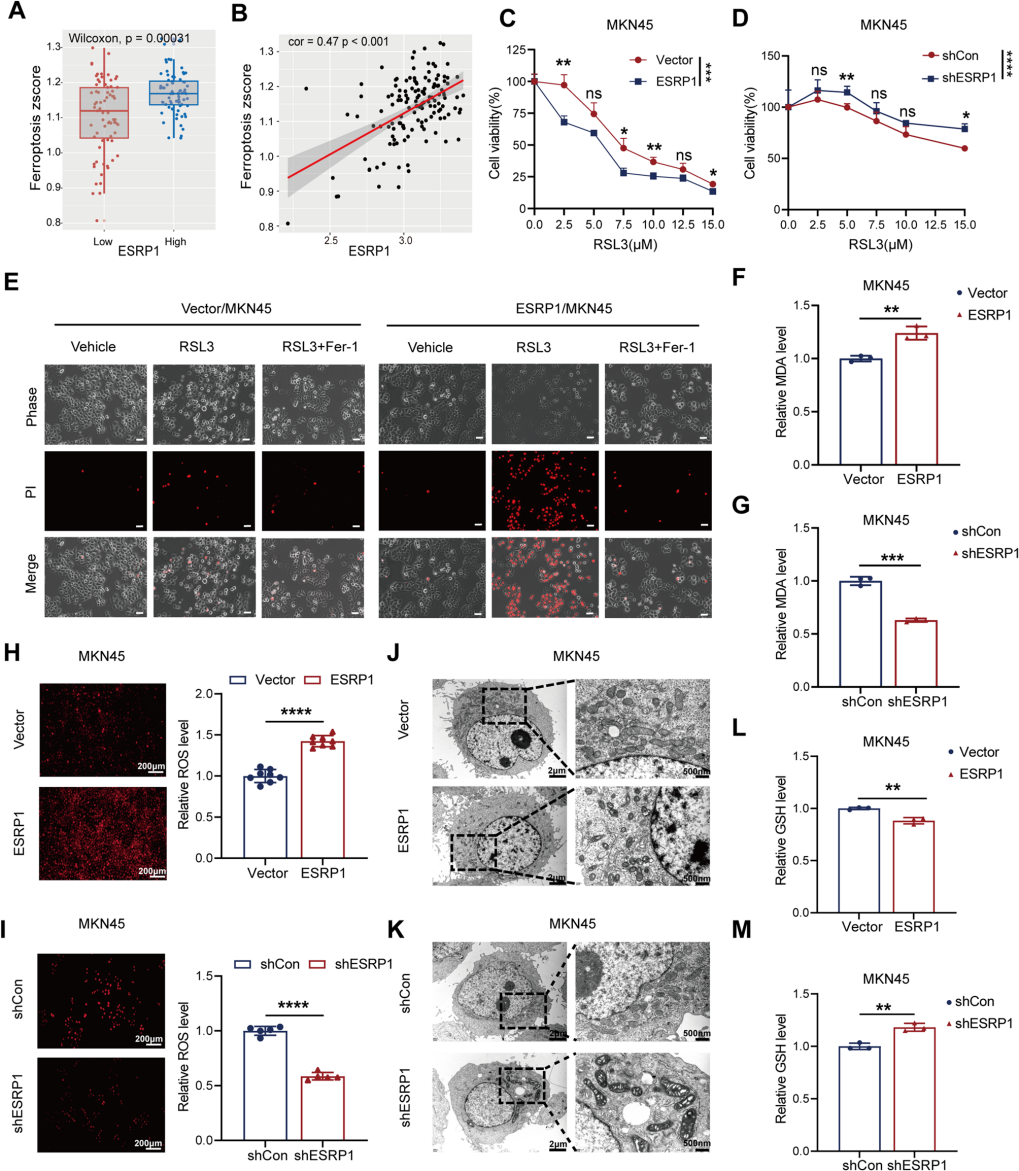
ESRP1 enhances sensitivity to ferroptosis of DGC cells. (**A**, **B**) ESRP1 is positively correlated with ferroptosis score. (**C**) Cell viability of MKN45/ESRP1 and MKN45/Vector sublines after RSL3 treatment as indicated. (**D**) Cell viability of MKN45/shESRP1 and MKN45/shCon sublines after RSL3 treatment as indicated. (**E**) Microscopy shows cell death. Cells were treated with RSL3(10 µM) for 24 h following pretreatment with or without Fer-1 (10 µM) for 6 h (scale bar: 100 μm). (**F**) The MDA assay kit was used to detect the relative MDA level of MKN45/ESRP1 and MKN45/Vector cells. (**G**) The MDA assay kit was used to detect the relative MDA level of shCon and shESRP1 transfected MKN45 cells. (**H**) Fluorescence microscope was used to measure intracellular ROS levels of MKN45/ESRP1 and MKN45/Vector cells (scale bar: 200 μm). (**I**) Fluorescence microscope was used to measure intracellular ROS levels of MKN45/shESRP1 and MKN45/shCon cells (scale bar: 200 μm). (**J**) Morphological changes of MKN45/ESRP1 and MKN45/Vector cells after RSL3 treatment as indicated (left scale bar: 2 μm; right scale bar: 500 nm). (**K**) Morphological changes of MKN45/shESRP1 and MKN45/shCon sublines after RSL3 treatment as indicated (left scale bar: 2 μm; right scale bar: 500 nm). (**L**)The GSH Assay Kit was used to measure intracellular GSH levels of MKN45/ESRP1 and MKN45/Vector cells. (**M**) The GSH Assay Kit was used to measure intracellular GSH levels of shCon or shESRP1 transfected MKN45 cells. The error bars indicate the mean ± SD. ns, no significance, *p < 0.05, **p < 0.01, ***p < 0.001, ***p < 0.0001

Previous studies [44–47] have demonstrated that fatty acid metabolism, glutathione and several metabolic processes in mitochondria have important roles in triggering ferroptosis. In our previous analysis, we found that ESRP1 was closely associated with unsaturated fatty acid metabolism, glutathione metabolism and the citric acid cycle. Therefore, we further explored the association between ESRP1 and ferroptosis signature. Further analysis indicated that ESRP1-high group had higher ferroptosis score (Fig. 5A) and ESRP1 expression was positively correlated with ferroptosis (Fig. 5B). Next, we examined the effect of ESRP1 expression on the ferroptosis-related phenotypes of DGC cells. Firstly, cell viability assays demonstrated that overexpression of ESRP1 in MKN45 increased sensitivity to RSL3-induced ferroptosis (Fig. 5C), while knockdown ESRP1 increased ferroptosis resistance (Fig. 5D). Additionally, propidium iodide (PI) staining revealed that RSL3 treated ESRP1-overexpression cells exhibited more cell deaths, which were significantly reversed by Fer-1 (ferroptosis inhibitor), suggesting that overexpression of ESRP1 sensitized DGC cells (Fig. 5E). As ferroptosis can be indicated by increased lipid peroxidation (malondialdehyde, MDA) and ROS level, we found that up-regulated ESRP1 increased the MDA level (Fig. 5F) and cellular ROS level (Fig. 5H), while down-regulated ESRP1 had the opposite effect (Fig. 5G, I). Furthermore, ferroptosis is morphologically characterized by the presence of smaller mitochondria exhibiting condensed mitochondrial membrane densities, accompanied by the reduction or complete absence of mitochondrial cristae, as well as the rupture of the outer mitochondrial membrane. As shown in Fig. 5J, RSL3 treatment induced the typical morphological alterations of mitochondria, indicative of ferroptosis occurrence, whereas ESRP1 knockdown alleviated these RSL3- induced mitochondrial changes in MKN45 cells (Fig. 5K). In addition, we also examined the effect of ESRP1 on glutathione, and the results showed that GSH levels decreased after overexpression of ESRP1(Fig. 5L), but increased after knockdown of ESRP1(Fig. 5M). Overall, these experimental findings support the significant role of ESRP1 in the regulation of ferroptosis in DGC.

### DHCR7 is the main downstream target of ESRP1

**Fig. 6:**
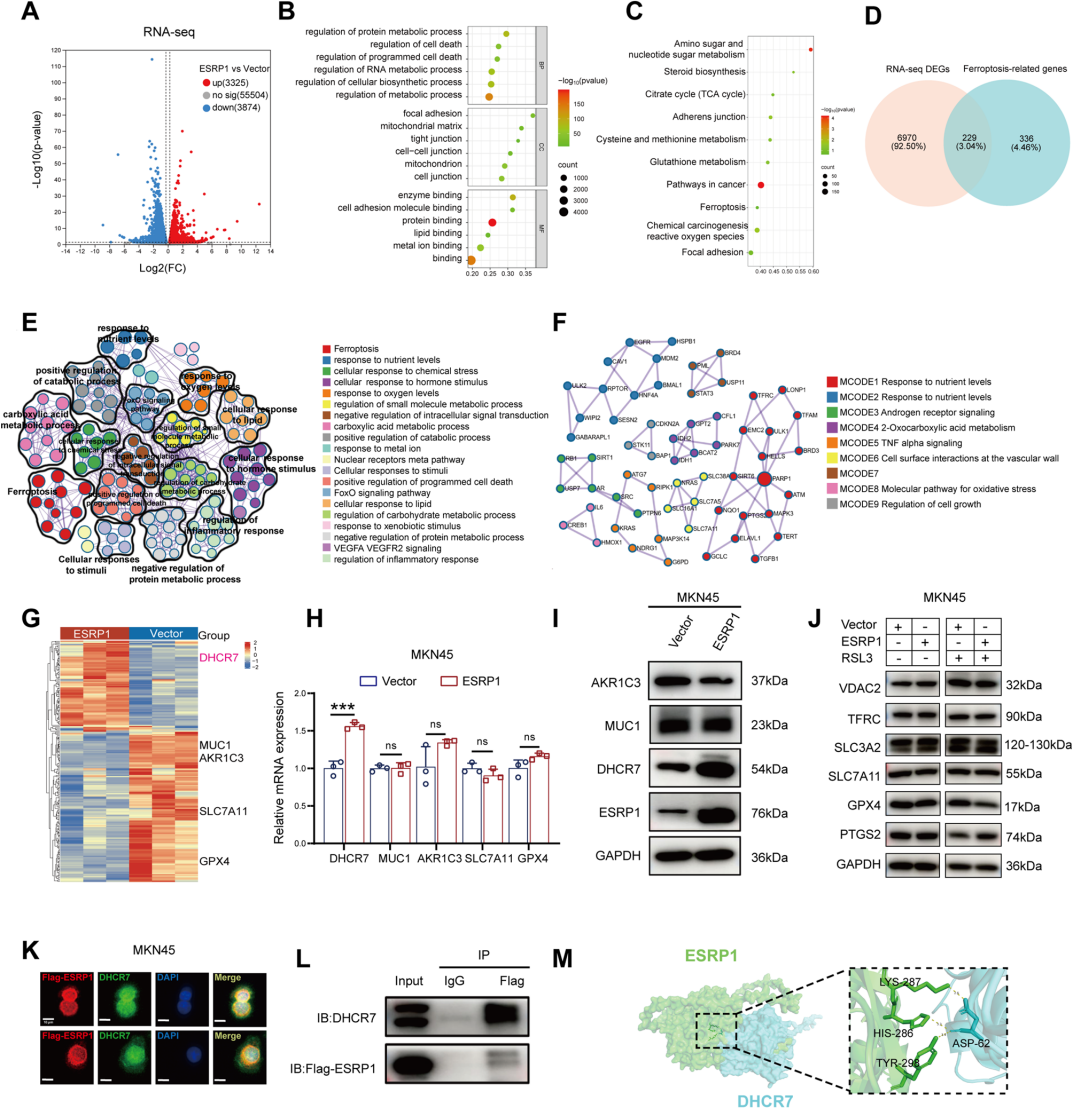
ESRP1 interacts with DHCR7. (**A**) Volcano plot shows DEGs based on RNA-seq in the overexpression-ESRP1 group compared with the control group in MKN45 cells. (**B**) GO enrichment analysis of DEGs. (**C**) KEGG enrichment analysis of DEGs. (**D**) 229 ferroptosis-related genes were identified from the overlap of genes identified by RNA-seq and FerrDb. (**E**) Metascape enrichment analysis based on the 229 ferroptosis-related genes. (**F**) PPI enrichment analysis by Metascape was performed to validate the relationship of identified 229 genes. (**G**) Heatmap of 229 ferroptosis-related genes in MKN45/ESRP1 and MKN45/Vector cells. (**H**) Quantitative real-time PCR analysis of mRNA expression levels of ferroptosis-related genes in MKN45/ESRP1 and MKN45/Vector cells. (**I**) Western blotting analysis of ferroptosis-relate proteins expression levels in MKN45/ESRP1 and MKN45/Vector cells. (**J**) Western blotting revealed changes in ferroptosis-related proteins mediated by ESRP1 overexpression under RSL3(10 µM, 24 h). (**K**) The co-localization of ESRP1 and DHCR7 was detected using a confocal microscopy (scale bar: 10 μm). (**L**) Co-IP assays show the specific interaction of ESRP1 with DHCR7. (**M**) Protein-protein docking analysis was performed to predict the binding sites of ESRP1 and DHCR7. The error bars indicate the mean ± SD. ns, no significance, ***p < 0.001

To further explore the potential molecular mechanisms between ESRP1 and ferroptosis activity, we performed RNA-seq on cells overexpressing ESRP1 and the control group. In the ESRP1 overexpression cells, we identified 7199 DEGs compared with the control group, including 3325 upregulated genes and 3874 downregulated genes (Fig. 6A, Table S3, *p*<0.05,|log2FC|>0.263). GO and KEGG enrichment analysis revealed that ESRP1 is involved mostly in metabolic process, cell death, cell adhesion, TCA cycle, and ferroptosis process (Fig. 6B, C). Subsequently, we identified 565 ferroptosisrelated genes through ferroptosis public databases (FerrDb V2) and literature (Table S4), and intersected these 565 genes with 7199 DEGs obtained by the above RNA-seq, and finally obtained 229 overlapped genes (Fig. 6D, Table S5). To further clarify the biological implications of these genes, we performed enrichment analysis and found that these genes were involved mostly in ferroptosis, response to nutrient levels, cellular response to chemical stress, positive regulation of programmed cell death, cellular response to lipid, etc. (Fig. 6E). We also utilized the 229 genes and conducted protein-protein interaction (PPI) enrichment analysis using the Metascape tool. By applying the Molecular Complex Detection (MCODE) method, we identified 9 sub-clusters of proteins, which are depicted in Fig. 6F. Cluster MCODE1 included the proteins PARP1, TFRC, and PTGS2, which were associated with response to nutrient levels (GO:0031667), regulation of protein localization to nucleus (GO:1900180), and response to hypoxia (GO:0001666). Cluster MCODE8 included IL6, CREB1, and HMOX1, which were associated with molecular pathway for oxidative stress (WP5477), parasitic infection pathways (R-HSA-9824443), and leishmania infection (R-HSA-9658195). Cluster MCODE9 included CDKN2A, STK11, and BAP1, which were associated with regulation of cell growth (GO:0001558), positive regulation of protein localization (GO:1903829), and regulation of growth (GO:0040008). The remaining cluster MCODE were related to androgen receptor signaling, 2-Oxocarboxylic acid metabolism, and TNF alpha signaling, and so forth. In addition, we selected significantly different as well as ferroptosis marker genes from 229 overlapped genes for validation, including GPX4, SLC7A11, MUC1, AKR1C1, DHCR7(Fig. 6G). However, qPCR results showed that only DHCR7, a pro- ferroptosis gene, was significantly increased after overexpression of ESRP1(Fig. 6H). Western blotting analysis showed that DHCR7 protein expression was also increased in ESRP1 overexpression cells (Fig. 6I). And we also examined the effects of ESRP1 on the expression of other known ferroptosis-related proteins using Western blotting, no significant changes in these proteins were observed, however, overexpression of ESRP1 increased the expression of PTGS2 and decreased the expression of GPX4 when treated with the ferroptosis inducer RSL3(Fig. 6J). Next, the relationship between ESRP1 and DHCR7 was further verified. Immunofluorescence experiments demonstrated the co-localization of ESRP1 and DHCR7 in the cell nucleus (Fig. 6K), and CoIP experiments were performed to verify the interaction between ESRP1 and DHCR7 (Fig. 6L). Subsequently, we used the protein-protein docking approach to predict the binding domain between ESRP1 and DHCR7 to gain a deeper understanding of their interaction (Fig. 6M). The above results suggest that DHCR7 may be a downstream target molecule of ESRP1.

### ESRP1 promotes ferroptosis activity and alleviates DGC progression by regulating DHCR7

**Fig. 7:**
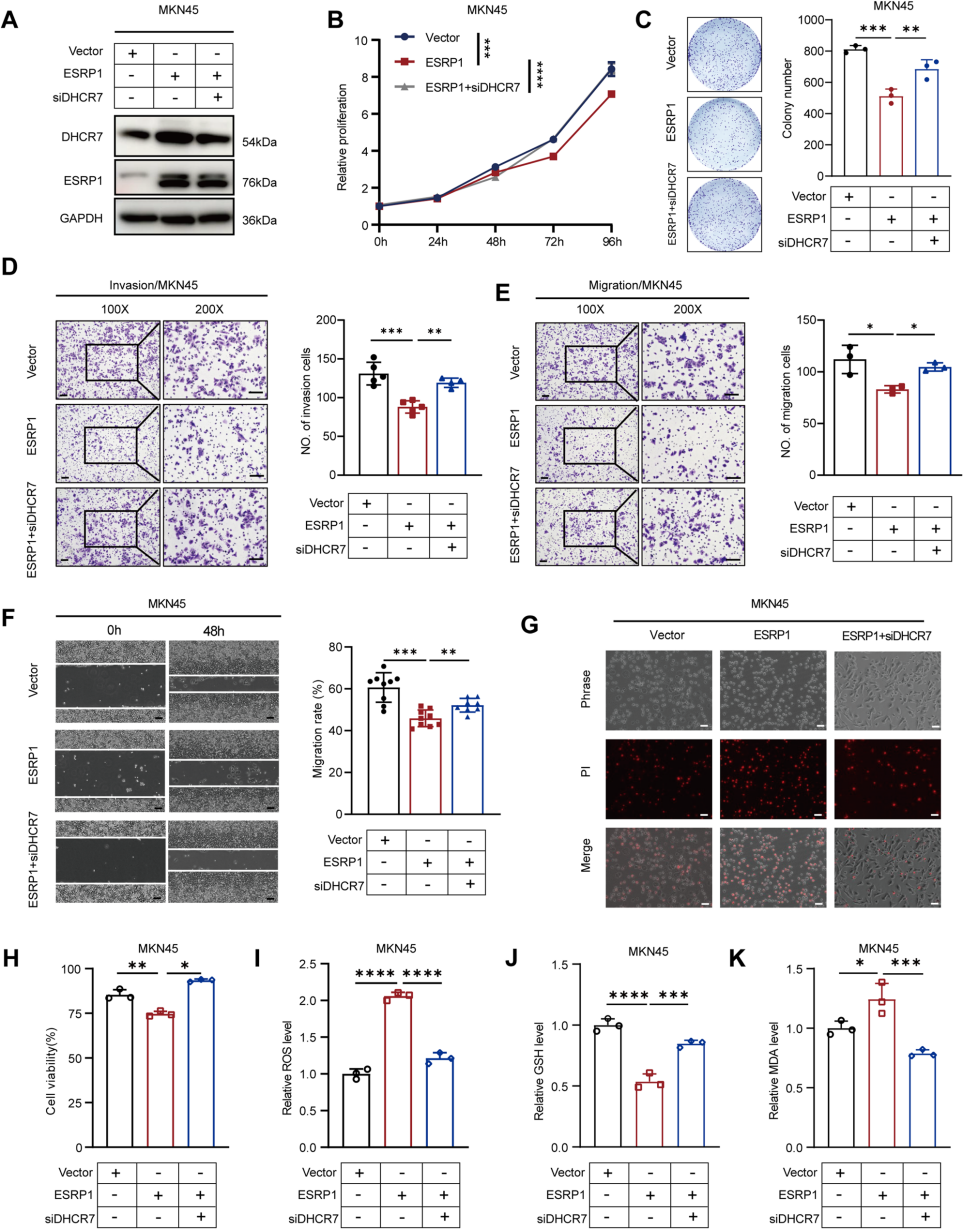
DHCR7 is the functional downstream protein of ESRP1. (**A**) Western blotting analysis of DHCR7 expression levels. (**B**) CCK-8 assays were performed to detect the proliferation ability. (**C**) Colony formation assays were performed to detect the colony formation ability. (**D**, **E**) Transwell assays were performed to detect the migration and invasion ability (left scale bar: 100 μm; right scale bar: 200 μm). (**F**) Wound healing assays were performed to detect the migration ability (scale bar: 100 μm). (**G**) Microscopy showing cell death (scale bar: 100 μm). (**H**) Cell viability of sublines after RSL3 treatment (10µM) as indicated. (**I**) The ROS levels were measured after ESRP1 overexpression and DHCR7 knockdown. (**J**) The GSH Assay Kit was used to measure intracellular GSH levels. (**K**) The MDA assay kit was used to detect the relative MDA level. The error bars indicate the mean ± SD. *p < 0.05, **p < 0.01, ***p < 0.001, ****p < 0.0001

Next, we performed resume experiments to clarify whether ESRP1 regulates ferroptosis and inhibits DGC progression by upregulating DHCR7 expression. The knockdown efficiencies of DHCR7 were verified by western blotting (Fig. 7A). Knockdown of DHCR7 rescued the reduced proliferation of DGC cells due to overexpression of ESRP1 (Fig. 7B), and also rescued the clone-forming ability of ESRP1 on gastric cancer cells (Fig. 7C). Overexpression of ESRP1 significantly inhibited the migration and invasion ability of DGC cells compared to control cells containing empty vector. However, knockdown of DHCR7 reversed the ESRP1-mediated effects on metastasis of DGC cells (Fig. 7D-F). Additionally, knockdown of DHCR7 rescued the effect of cell death (Fig. 7G), cell viability (Fig. 7H), ROS level (Fig. 7I), GSH level (Fig. 7J) and MDA (Fig. 7K) due to overexpression of ESRP1. In summary, these results suggest that ESRP1 promotes ferroptosis activity and inhibits the malignant progression of DGC by upregulating DHCR7 expression.

### Prediction and screening of small-molecule drugs

Furthermore, we explored the relationship between ESRP1 expression and drug sensitivity. The necessity of screening ESRP1 for GC using RNAi and CRISPR-Cas9 techniques from DepMap database. Median CERES score was below 0.2, which means the gene is an essential gene. And most of RNAi score were below 0 (RNAi approach to 0 means the gene is not an essential gene, Fig. S5). These findings indicated that ESRP1 could be regarded as a crucial gene in GC.

**Fig. 8:**
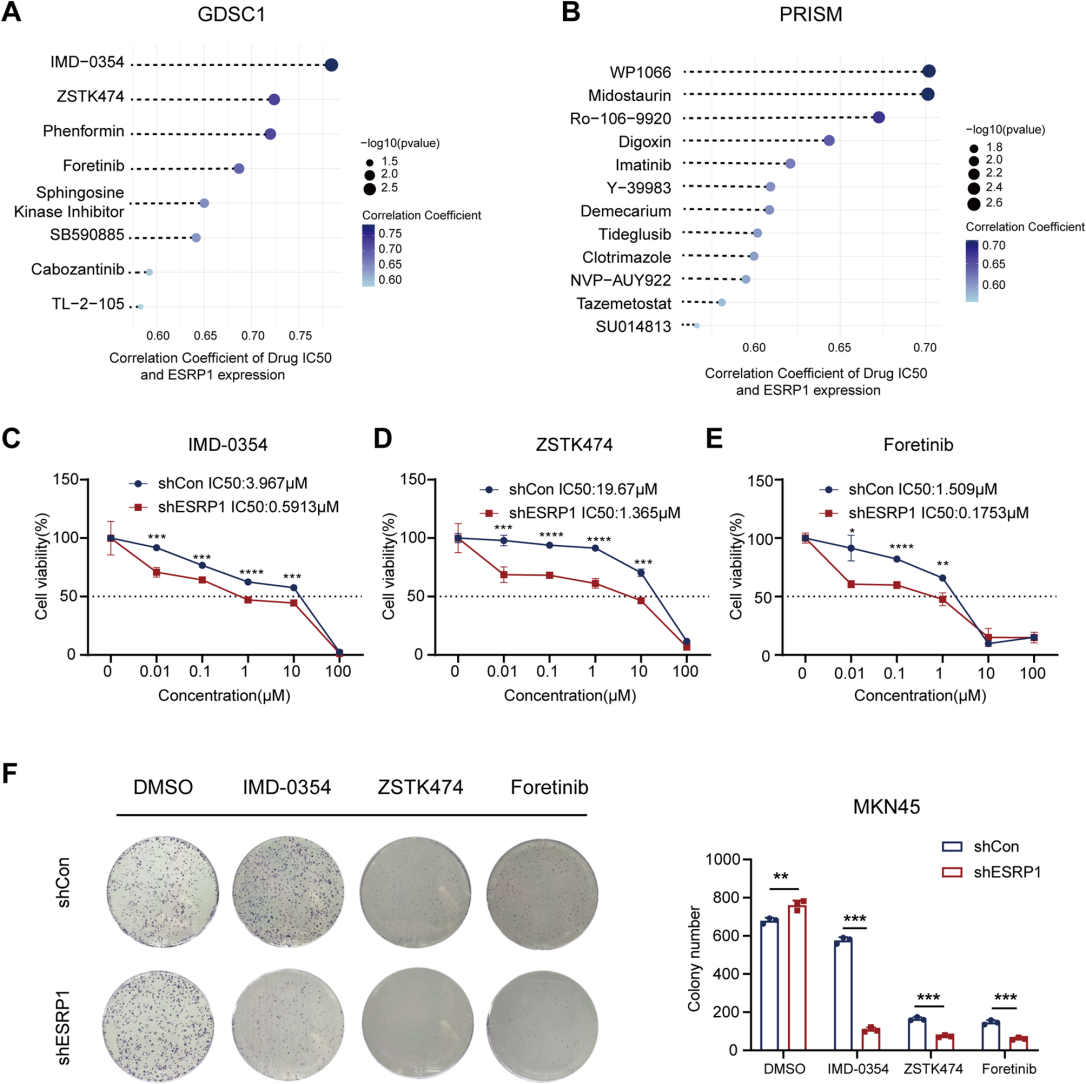
Prediction of small molecule drugs associated with ESRP1. (**A**) Correlation between drug IC50 and ESRP1 expression in GDSC1. (**B**) Correlation between drug IC50 and ESRP1 expression in PRISM. Cell viability assays of IMD-0354 (**C**), ZATK474 (**D**), and Foretinib (**E**) at concentrations as indicated for 24 h in the shESRP1 and shCon groups. (**F**) Colony-formation assays of shCon/MKN45 and shESRP1/MKN45 treated with DMSO or three drugs as indicated. The error bars indicate the mean ± SD. *p < 0.05, **p < 0.01, ***p < 0.001, ****p < 0.0001

Subsequently, we applied two drug response databases (PRISM and GDSC) to predict chemotherapy response of the small-molecule drugs for DGC patients with ESRP1-low expression. Eight drugs exhibited positive correlation with ESRP1 (indicating more sensitive to ESRP1-low subgroup) from GDSC portal, such as IMD.0354 (IKKβ inhibitor), ZSTK474 (PI3K inhibitor), Phenformin, Foretinib (c-Met inhibitor), Sphingosine Kinase Inhibitor, SB590885 (BRAF inhibitor), Cabozantinib (VEGFR and MET inhibitor), and TL-2-105 (Fig. 8A, Fig. S6A, and Table S6). In addition, we identified 12 small-molecule drugs positively correlated with ESRP1 from PRISM databases, including WP1066 (STAT inhibitor), Midostaurin (FLT3 inhibitor, KIT inhibitor, PKC inhibitor), Ro.106.9920 (NF-κB inhibitor), Digoxin (Na^+^-K^+^- ATPase inhibitor), Imatinib (multi-target tyrosine kinase inhibitors), Y.39,983 (ROCK inhibitor), Demecarium (Acetylcholinesterase inhibitor), Tideglusib (Glycogen synthase kinase inhibitor), Clotrimazole(Cytochrome P450 inhibitor), NVP-AUY922 (HSP inhibitor), Tazemetostat (Histone lysine methyltransferase inhibitor), and SU14813(VEGFR inhibitor) (Fig. 8B, Fig. S6B, and Table S7). To further investigate the relationship between ESRP1 expression and drug sensitivity, we selected several small molecule drugs with high correlation coefficients in the GDSC and PRISM databases for experimental validation. The results of CCK-8 and clone formation assays revealed that, after treatment with IMD-0354, ZSTK474, and Foretinib, ESRP1-low cells showed reduced viability and suppressed colony formation compared to ESRP1-high cells (Fig. 8C-F). However, the drug sensitivities of WP1066 and Midostaurin did not show significant difference in the ESRP1 subgroups (Fig. S7A-C), suggesting that DGC cells in the knockdown ESRP1 group were more sensitive to the three drugs (IMD-0354, ZSTK474, and Foretinib). The three potential small-molecule drugs were significantly positively correlated with the ESRP1 and may have potential therapeutic implications for patients with ESRP1-low expression in DGC.

## Discussion

In this study, we conducted a thorough bioinformatics analysis to investigate the correlation between ESRP1 expression levels and survival outcomes in DGC. Our findings indicated that a higher expression of ESRP1 was associated with a better prognosis in DGC, highlighting its potential as a prognostic biomarker. Additionally, our data revealed a close relationship between ESRP1 expression and ferroptosis in DGC, providing new insights into the critical function of ESRP1. Clinically, ESRP1 expression levels could stratify patients into high- and low-expression subgroups, guiding personalized treatment strategies. For patients with high ESRP1 expression, ferroptosis-inducing agents may enhance therapeutic efficacy, as ESRP1 sensitizes DGC cells to ferroptosis via DHCR7 upregulation. Conversely, for ESRP1-low patients, our identified small-molecule inhibitors (IMD-0354, ZSTK474, and Foretinib) could serve as alternative therapies. Overall, our research sheds light on the potential of ESRP1 as a therapeutic target and prognostic biomarker for DGC.

ESRP1 is an epithelial cell-specific RNA-binding protein that regulates the alternative splicing of multiple genes involved in epithelial mesenchymal transition (EMT), which plays a critical role in metastasis by reducing tumor motility and invasiveness [48, 49]. Studies have reported that ESRP1 plays a crucial role in suppressing tumorigenic potential and/or attenuating metastasis in various types of cancer [18, 20, 50, 51]. For instance, relevant studies have shown that ESRP1 overexpression reduces the migration and proliferation of pancreatic cancer cells in vitro, and rarely produces liver metastases in vivo [50]. Furthermore, clinical data showed that ESRP1 expression levels were positively correlated with patient survival in clear cell renal cell carcinoma and breast cancer [52]. Although accumulating evidence indicates the roles and clinical significance of ESRP1 in tumor progression and metastasis, the role and regulatory mechanisms of ESRP1 in gastric cancer, especially in DGC, have not been thoroughly studied. Our study revealed that ESRP1 expression was positively correlated with prognosis of DGC, and patients with high expression of ESRP1 had a better prognosis than those with low expression of ESRP1. Interestingly, we only observed this relationship between ESRP1 and prognosis in diffuse gastric cancer, but did not observe the relationship in intestinal and mixed gastric cancer. To identify the role of ESRP1 in regulating the invasiveness and motility of diffuse-gastric cancer cells, we conducted Transwell migration and Matrigel invasion assays using MKN45 cells. Overexpression of ESRP1 significantly decreased the migration and invasion of MKN45 cells and animal experiments have further confirmed the ability of ESRP 1 to hinder the metastasis of DGC. These results supported the notion that ESRP1 has a critical function in metastasis and tumorigenesis by suppressing tumor motility and invasiveness in DGC.

Ferroptosis is induced by the accumulation of lipid peroxidation products, and the key factor in ferroptosis execution is iron-catalyzed peroxidation of polyunsaturated fatty acid-containing phospholipids (PUFA-PLs). This can result in the lethal accumulation of lipid peroxides on cellular membranes, leading to membrane rupture and subsequent ferroptosis [53]. Additionally, ferroptosis requires the citric acid cycle and various anaplerotic reactions that fuel it in mitochondria, which drive ferroptosis likely through the promotion of reactive oxygen species (ROS), ATP and/or PUFA-PL generation [54, 55]. In our study, we found that ESRP1 was positively associated with unsaturated fatty acid metabolism and citric acid cycle. Therefore, we speculated that ESRP1 may regulate the progression of diffuse gastric cancer through the ferroptosis. Furthermore, we explored the association between ESRP1 and ferroptosis signature and found that ESRP1 was closely associated with ferroptosis. Our results showed that DGC cells with ESRP1 high expression were more sensitive to ferroptosis. Previous studies have demonstrated that ESRP dysregulation contributes to cancer progression by regulating EMT, drug resistance, and metabolic reprogramming [17, 56]. In this study, we identified a novel role for ESRP1 in DGC cells that impacts tumor progression through the regulation of ferroptosis processes.

Mechanistically, our study has shown that ESRP1 has the potential to induce ferroptosis by increasing the expression of 7-dehydrocholesterol reductase (DHCR7), an enzyme that catalyzes cholesterol synthesis and acts in the final step of the cholesterol biosynthesis pathway, converting 7-dehydrocholesterol (7-DHC) into cholesterol [57]. Recent studies have reported that DHCR7 is a pro-ferroptotic gene, and its substrate 7-DHC is an endogenous inhibitor of ferroptosis [58]. Florencio Porto Freitas et al. used CRISPR/Cas9-mediated genome-wide screening to identify DHCR7 as a regulator of ferroptosis in hepatocytes, and inhibition of DHCR7 can reduce ferroptosis of human hepatocellular carcinoma cells [58]. Therefore, DHCR7 and 7-DHC may be potential targets for tumor treatment. Our study has revealed that ESRP1 can interact with DHCR7, and up-regulation of ESRP1 will increase the expression of DHCR7. The rescue experiments proved that knockdown of DHCR7 could be restored the effect of ESRP1 overexpression on proliferation, migration, invasion and ferroptosis of gastric cancer cells, indicating that ESRP1 inhibits DGC progression by promoting DHCR7-mediated ferroptosis.

The study’s limitation was the use of available datasets from various cohorts that differed in patients’ origin and data processing. Moreover, we only analyzed data from public databases and lacked validation from clinical samples. In addition, the mechanistic relationship between ESRP1 and DHCR7 needs further exploration. The ESRP1-DHCR7 axis warrants deeper investigation, particularly its role in lipid metabolism. Therefore, further validation is necessary to establish the relationship between biological processes and ESRP1 expression, including an analysis of metabolic reprogramming and ferroptosis. In the future, it is necessary to integrate single-cell sequencing, metabolomics, and spatial transcriptomics to analyze the molecular map of DGC ferroptosis from the multi-omics level, promote the development of synergistic strategies of ferroptosis inducers and targeted therapy and immunotherapy, and provide innovative personalized solutions for improving the prognosis of DGC patients.

## Conclusion

In conclusion, our findings revealed that ESRP1 is a novel tumor suppressor related to ferroptosis in DGC. Increased ESRP1 expression was significantly associated with favorable survival outcomes. These new findings provide insight of ESRP1 into its functions as a novel independent prognostic factor, and a new potential target for improving the prognosis of DGC patients.

## Electronic supplementary material

Below is the link to the electronic supplementary material.

Supplementary Material 1

Supplementary Material 2

## Data Availability

No datasets were generated or analysed during the current study.

## Abbreviations

GC: Gastric cancer
DGC: Diffuse-type gastric cancer
IGC: Intestinal-type gastric cancer
ESRP1: Epithelial splicing regulatory protein 1
DHCR7: 7-dehydrocholesterol reductase
EMT: Epithelial mesenchymal transition
TCGA: The Cancer Genome Atlas
ACRG: Asian Cancer Research Group
KUGH: Korea University Guro Hospital
KUCM: Kosin University College of Medicine
BJCH: Beijing Cancer Hospital
CPTAC: Clinical Proteomic Tumor Analysis Consortium
DEGs: Differentially expressed genes
GO: Gene Ontology
KEGG: Kyoto encyclopedia of genes and genomes
CNV: Copy number variation
TMB: Tumor mutation burden
IHC: Immunohistochemistry
PI: Propidium Iodide
ROS: Reactive oxygen species
MDA: Malondialdehyde
GSH: Glutathione
TEM: Transmission Electron Microscope
Co-IP: Co-immunoprecipitation
