# Supplementary material for: ESRP1 exerts anti-tumor role by promoting ferroptosis in diffuse-type gastric cancer
Source: J Exp Clin Cancer Res. 2025 Jun 18;44:176. doi: 10.1186/s13046-025-03435-2 (PMC12175430; doi:10.1186/s13046-025-03435-2)
Supplement: Supplementary file 1 — Supplementary Material 1 [file 13046_2025_3435_MOESM1_ESM.docx]

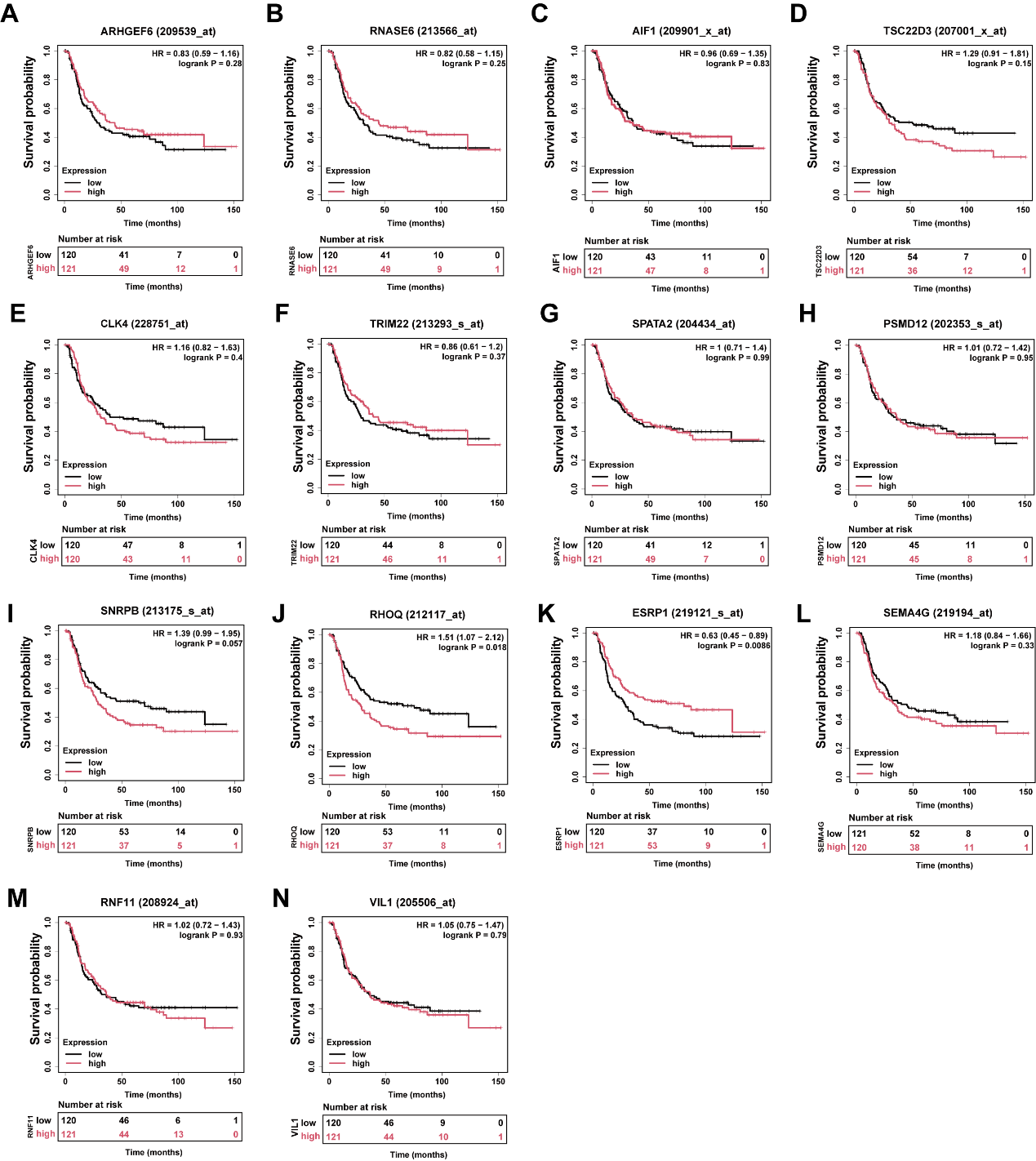


**Fig. S1:** Kaplan-Meier survival curves of overall survival based on 14 DEGs median expression in patients with DGC. Correlation between the expression of ARHGEF6(A), RNASE6(B), AIF1(C), TSC22D3(D), CLK4(E), TRIM22(F), SPATA2(G), PSMD12(H), SNRPB(I), RHOQ(J), ESRP1(K), SEMA4G(L), RNF11(M), VIL1(N) and OS in DGC patients were analyzed by the Kaplan-Meier Plotter.


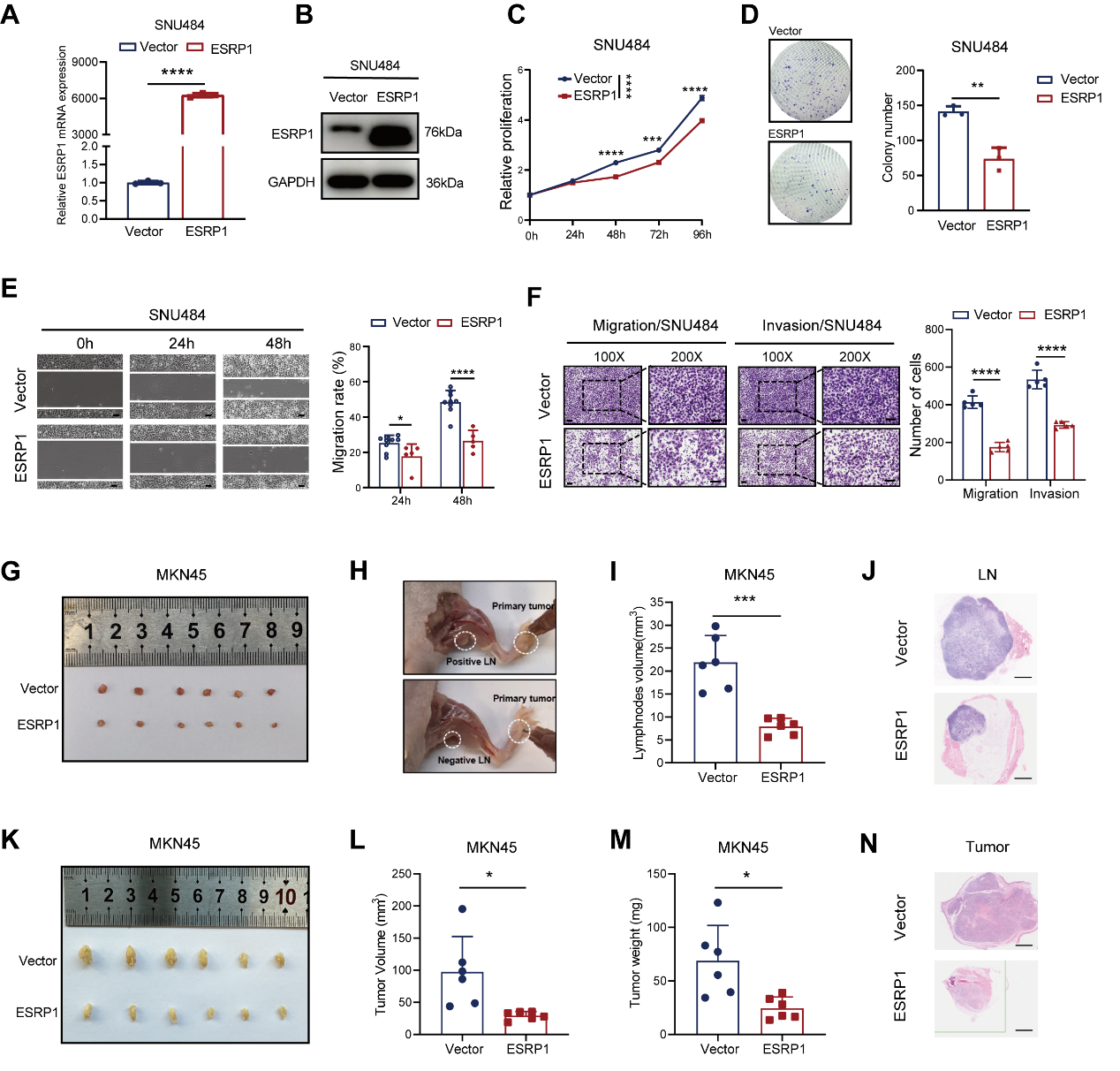


**Fig. S2:** Overexpression of ESRP1 inhibits the malignant progression in DGC cells. (A) The overexpression efficiencies of ESRP1 in mRNA level were detected by Quantitative real-time PCR (qRT-PCR). (B)The overexpression efficiencies of ESRP1 in protein level were detected by Western blotting. (C) Cell proliferation was detected by CCK-8 assay in ESRP1-overexpresion SNU484 cell lines and negative control group. (D) Representative images and the quantification of colony numbers of SNU484 cells transfected with the vector (vector control) and ESRP1 (ESRP1 overexpression). (E) Representative images and the quantification of migration rate from wound healing assays performed with the ESRP1 overexpression cells and control group (scale bar: 100μm). (F) Representative data from Transwell migration and Matrigel invasion assays performed with the ESRP1 overexpression SUN484 cells and control group (left scale bar: 100 μm; right scale bar: 200 μm). (G) Representative image of popliteal lymph nodes of mice injection with ESRP1 overexpression and the negative control MKN45 cells (n = 6). (H) Representative images of popliteal lymph nodes metastasis and non-metastasis mice. (I) The volume of popliteal lymph nodes in each group (n = 6). (J) H&E staining of popliteal lymph nodes (scale bar: 1 mm). (K) Representative anatomy image of footpad tumor of mice injection with ESRP1 overexpression and the negative control MKN45 cells (n = 6). (L) The footpad tumor volume in each group (n = 6). (M) The footpad tumor weight in each group (n = 6). (N) H&E staining of footpad tumor (scale bar: 1 mm). The error bars indicate the mean ± SD. *p < 0.05, **p < 0.01, ***p < 0.001, ****p < 0.0001.


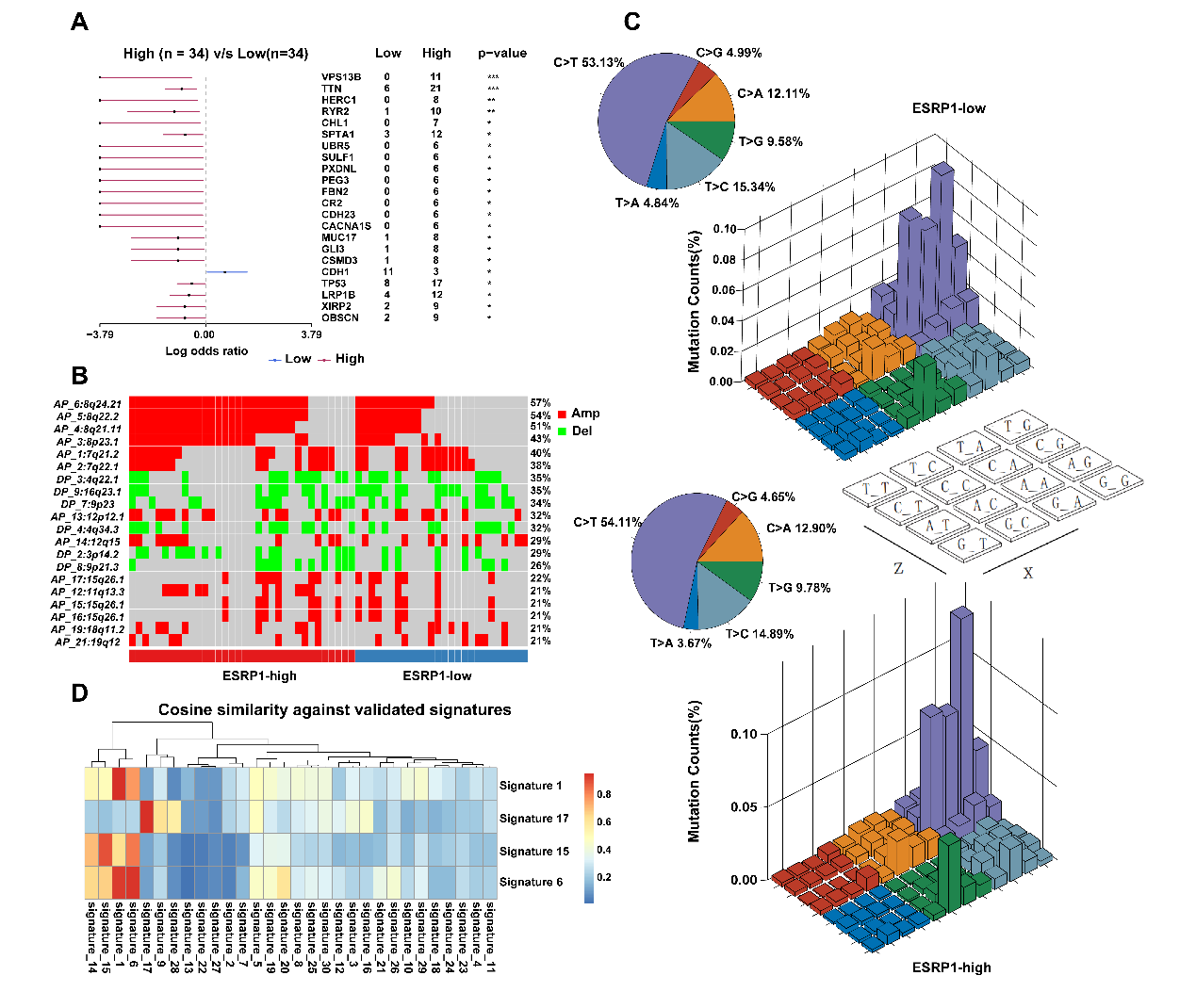


**Fig. S3:** Comparison of genomic alterations between ESRP1-low expression group and -high expression group in the TCGA cohort. (A) Forest plot of the gene mutation frequency associated with ESRP1 expression level. *P*-value was calculated by Fisher’s exact test. (B)Heatmap of gene amplification and deletion in two groups. (C) Lego plot representation of mutation patterns in 68 samples. Single-nucleotide substitutions were divided into six categories with 16 surrounding flanking bases. The pie chart showed the proportion of six categories of mutation patterns. (D)Cosine similarity analysis of extracted mutational signatures against the 30 identified signatures in Catalogue of Somatic Mutations in Cancer (COSMIC, v2) with heatmap illustration. **p* < 0.05, ***p* < 0.01, ****p* < 0.001.


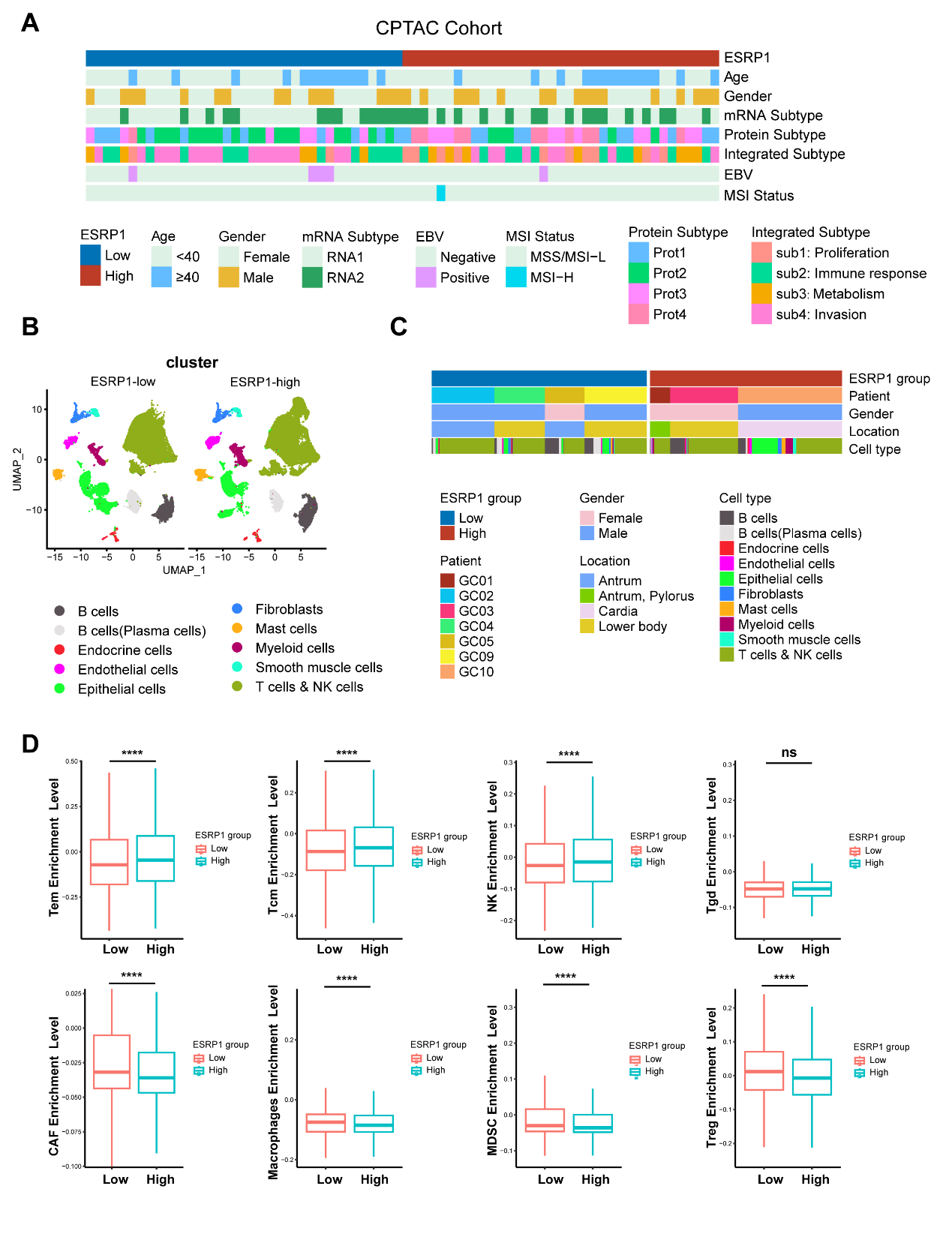


**Fig. S4:** Comparison of clinical characteristics and molecular processes. (A) The different clinical features and molecular subtypes in the CPTAC cohort with ESRP1 subgroup. (B) UMAP plots display 104,028 cells derived from scRNA-seq, clustered into ten major cell types. (C) The distribution of the tumor sample, anatomical location, gender and single cell annotation among the bulk-seq identified ESRP1 different expression subgroups. (D) Comparison of cell infiltration level based on scRNA-seq between the ESRP1-low and ESRP1-high subgroups. *****p* < 0.0001.


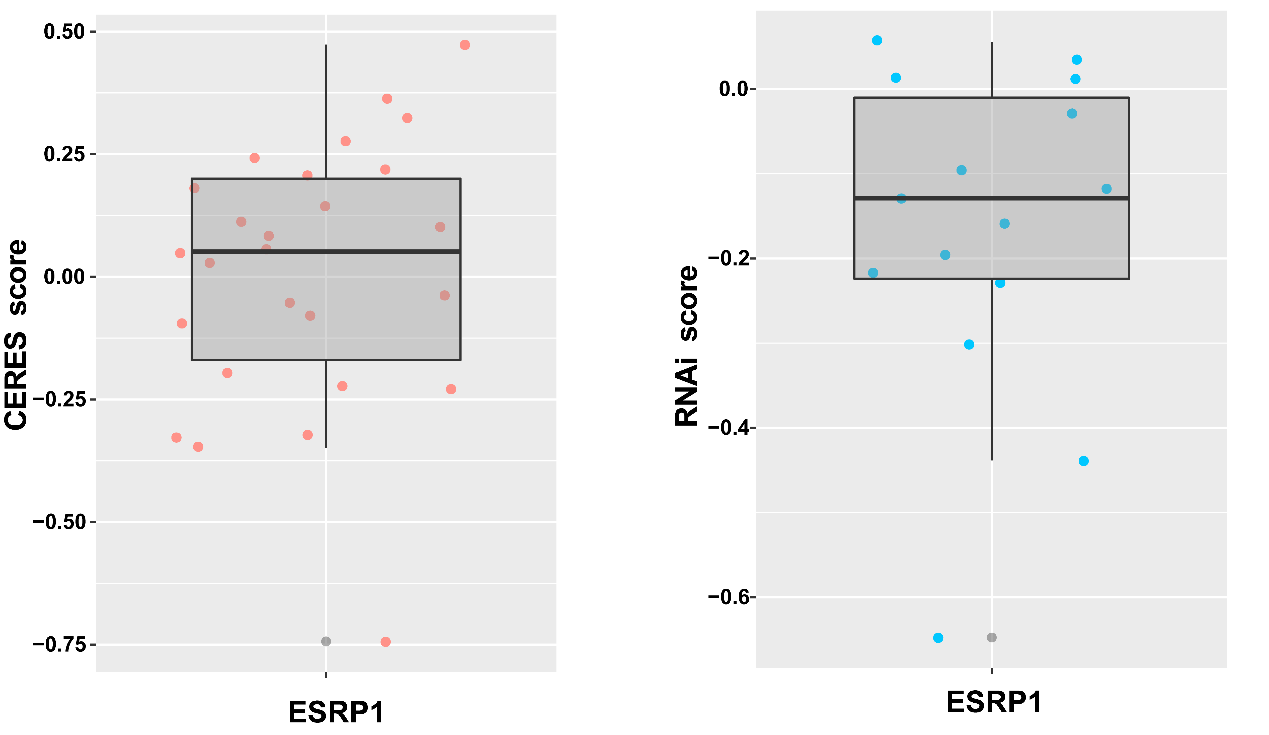


**Fig. S5:** The CERES score and RNAi score evaluated the necessarily of ESRP1 for diffuse gastric cancer cell survival, and the data were downloaded from the DepMap.


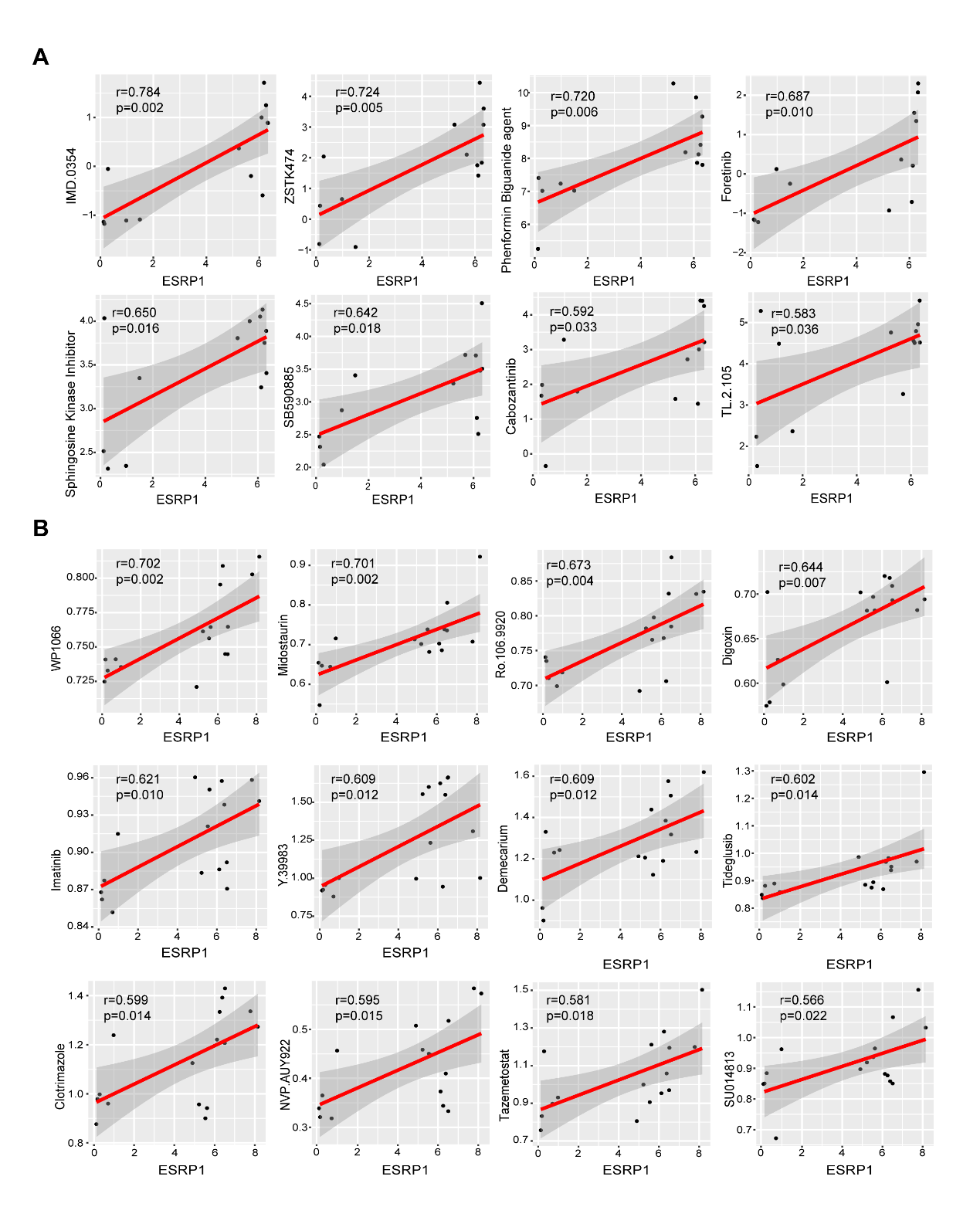


**Fig. S6:** Prediction of small molecule drugs associated with ESRP1. (A) Correlation between drug IC50 values and ESRP1 expression in the GDSC1. (B) Correlation between drug IC50 values and ESRP1 expression in the PRISM.

**
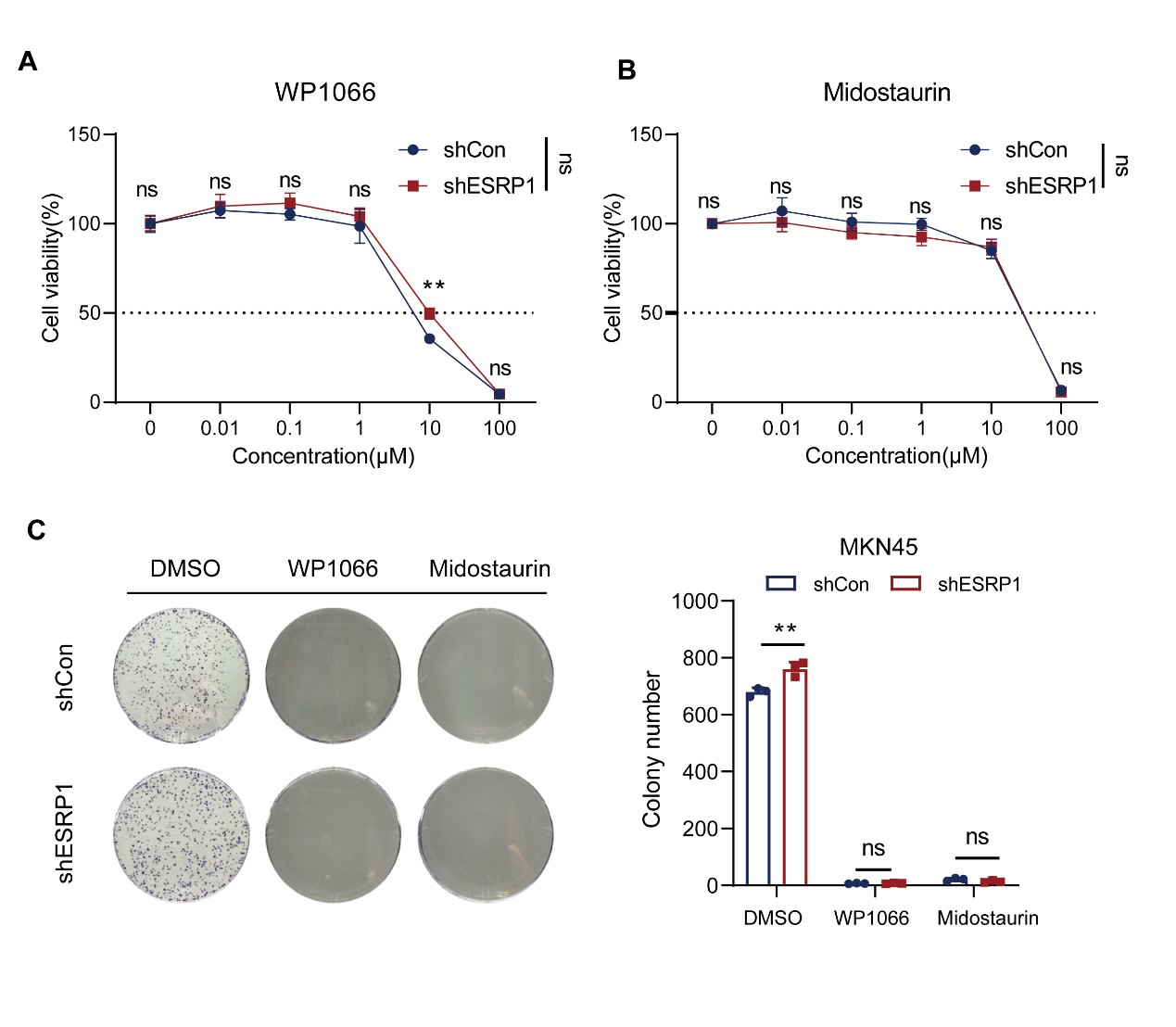
**

**Fig. S7:** Drug susceptibility analysis of WP1066 and Midostaurin. Cell viability assay of WP1066 (A) and Midostaurin (B) at concentrations as indicated for 24h in the shESRP1 and shCon groups. (C) Colony-formation assays of shCon/MKN45 and shESRP1/MKN45 treated with DMSO or two drugs as indicated. The error bars indicate the mean ± SD. ns, no significance, ns, no significance, *****p* < 0.0001.
